# Identification of IGF-1-enhanced cytokine expressions targeted by miR-181d in glioblastomas via an integrative miRNA/mRNA regulatory network analysis

**DOI:** 10.1038/s41598-017-00826-0

**Published:** 2017-04-07

**Authors:** Kuo-Hao Ho, Peng-Hsu Chen, Edward Hsi, Chwen-Ming Shih, Wei-Chiao Chang, Chia-Hsiung Cheng, Cheng-Wei Lin, Ku-Chung Chen

**Affiliations:** 1 Department of Clinical Pharmacy, School of Pharmacy, Taipei Medical University, Taipei, Taiwan; 2 Graduate Institute of Medical Sciences, College of Medicine, Taipei Medical University, Taipei, Taiwan; 3 Graduate Institute of Biomedical Sciences, China Medical University, Taichung, Taiwan; 4 Department of Biochemistry and Molecular Cell Biology, School of Medicine, College of Medicine, Taipei Medical University, Taipei, Taiwan; 5 Department of Clinical Pharmacy, Master Program for Clinical Pharmacogenomics and Pharmacoproteomics, School of Pharmacy, Taipei Medical University, Taipei, Taiwan; 6 Department of Pharmacy, Taipei Medical University-Wanfang Hospital, Taipei, Taiwan

## Abstract

The insulin-like growth factor (IGF)-1 signaling is relevant in regulating cell growth and cytokine secretions by glioblastomas. MicroRNAs determine the cell fate in glioblastomas. However, relationships between IGF-1 signaling and miRNAs in glioblastoma pathogenesis are still unclear. Our aim was to validate the IGF-1-mediated mRNA/miRNA regulatory network in glioblastomas. Using *in silico* analyses of mRNA array and RNA sequencing data from The Cancer Genome Atlas (TCGA), we identified 32 core enrichment genes that were highly associated with IGF-1-promoted cytokine-cytokine receptor interactions. To investigate the IGF-1-downregulated miRNA signature, microarray-based approaches with IGF-1-treated U87-MG cells and array data in TCGA were used. Four miRNAs, including microRNA (miR)-9-5p, miR-9-3p, miR-181d, and miR-130b, exhibited an inverse correlation with IGF-1 levels. The miR-181d, that targeted the most IGF-1-related cytokine genes, was significantly reduced in IGF-1-treated glioma cells. Statistical models incorporating both high-IGF-1 and low-miR-181d statuses better predicted poor patient survival, and can be used as an independent prognostic factor in glioblastomas. The C-C chemokine receptor type 1 (CCR1) and interleukin (IL)-1b demonstrated inverse correlations with miR-181d levels and associations with patient survival. miR-181d significantly attenuated IGF-1-upregulated CCR1 and IL-1b gene expressions. These findings demonstrate a distinct role for IGF-1 signaling in glioma progression via miR-181d/cytokine networks.

## Introduction

The tumor microenvironment is established by interactions between malignant and non-cancerous cells, including fibroblasts, immune cells, surrounding blood vessels, the extracellular matrix, and cytokines^1^. All tumor-associated cells and signaling molecules provide regulatory support for controlling tumor growth, angiogenesis, metastasis, and peripheral immune tolerance^2^. Among those, cytokines play vital roles in regulating cell-cell interactions, tumor progression, and antitumor immune responses. Cytokines, small molecules composed of glycoproteins and polypeptides, exert diverse functions depending on the microenvironment. A variety of cytokines display aberrant expression levels and functions in cancers, including in glioblastoma multiforme (GBM)^3^. GBM belongs to grade IV primary malignant gliomas with a poor prognosis and high lethality in adults^4^. Abnormally expressed cytokines such as interleukins (ILs), colony-stimulating factors, interferons (IFNs), tumor necrosis factor (TNF), transforming growth factor (TGF)-β, and other chemokines have been implicated in glioma progression^5^. Understanding the functional regulatory mechanisms of key cytokines could provide potential therapeutic applications and directions for GBM.

The insulin-like growth factor (IGF) signaling axis exhibits pleiotropic properties in promoting cellular proliferation, individual development, and progression of various diseases, including cancer^6^. Increasing evidence suggests that IGF-1 can influence cytokine secretions^7^. For example, IGF-1 modulates inflammatory cytokine and chemokine levels such as TNF-α and CC chemokines to accelerate muscle regeneration^8^. IGF-1 also regulates inflammatory responses in glioma cells via influencing HIF-1α-TLR9 crosstalk^9^. In contrast, TNF-α, IL-1α, and IL-6 significantly inhibit IGF-1-stimulated proteoglycan synthesis via repressing IGF-1 activity^10^. Since the IGF-1 signaling axis is recognized as a cancer therapeutic target^11^, clarifying relationships between IGF-1 stimulation and cytokine secretions in glioma progression is an important issue.

MicroRNAs (miR) are endogenous, small, non-coding RNAs that regulate gene expressions by binding to the 3′ untranslated region (UTR) of their target messenger (m)RNAs for degradation and/or translational repression. Aberrant miRNA expressions were identified in GBM development^12^. The miR-181d, an intergenic miRNA belonging to the miR-181 family (a, b, c, and d), forms a cluster gene with miR-181c on chromosome 19. By inhibiting K-ras and Bcl-2 expressions, miR-181d acts as a tumor suppressor in gliomas^13^. Since an inverse correlation was identified between miR-181d and methyl-guanine-methyl-transferase (MGMT) levels in GBM patients, miR-181d could also be a predictive biomarker for the response to temozolomide (TMZ)^14, 15^. However, no studies have mentioned relationships between miR-181d and cytokine expressions in GBM progression. Furthermore, the molecular mechanisms that influence miR-181d gene expression in GBM development are still unclear.

It is well established that both the IGF signaling axis and miRNA regulation are critical for glioma progression. However, no studies have reported IGF-mediated miRNA networks and functions in glioma cells. In the present study, we aimed to clarify relationships among IGF, miRNAs, and cytokine expressions in glioma development. By comprehensively analyzing transcriptomic profiles with a GBM microarray and RNA sequencing (Seq) data in The Cancer Genome Atlas (TCGA) database, we identified genes highly associated with IGF-1 levels and involved in cytokine-cytokine receptor interactions. An IGF-1-downregulated miRNA profile was obtained from miRNA array analyses with IGF-1-stimulated glioma U87-MG cells and TCGA database. By an integrative miRNA/mRNA regulatory network analysis, miR-181d showed the highest correlations with IGF-1-related cytokines. Finally, interleukin (IL)-1b and C-C chemokine receptor type 1 (CCR1), which is upregulated by IGF-1 stimulation, were identified as direct target genes of miR-181d. Taken together, these results demonstrate that IGF-1-inhibited miR-181d is involved in enhancing IL-1b and CCR1 cytokine expressions in GBM development.

## Results

### Identification of differentially expressed genes (DEGs) and pathways associated with the IGF-1 expression status in GBM patients of TCGA

**Figure 1:**
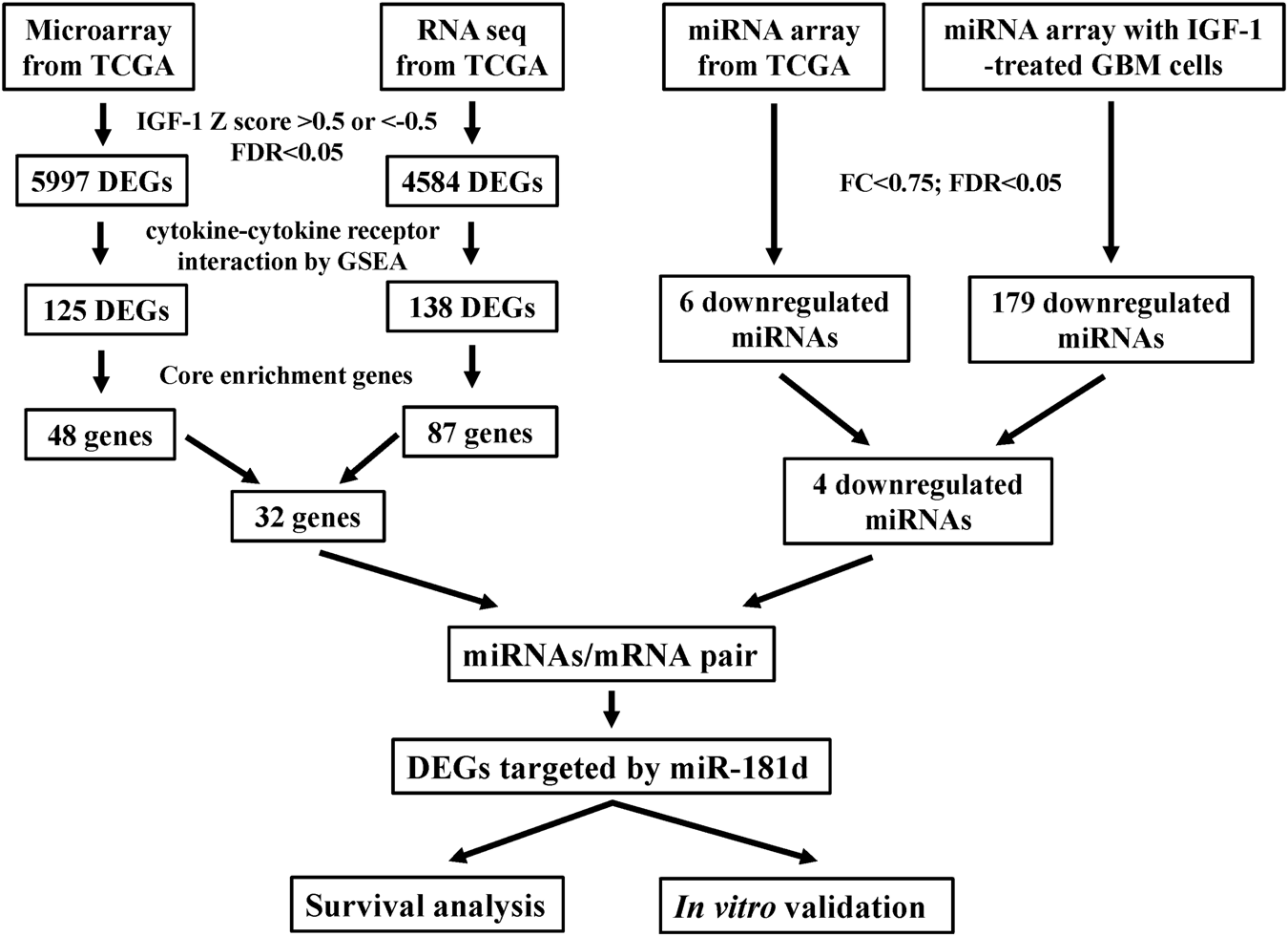
Flowcharts for analyzing the insulin-like growth factor (IGF)-1 mediated messenger (m)RNA/micro (mi)RNA networks in glioblastoma multiforme (GBM). DEGs, differentially expressed genes, FDR, false discovery rate; MC, multiple of change. MC, multiple of change.

The flowchart in Fig. 1 demonstrates the detailed processes of integrative analyses for exploring IGF-1-mediated miRNA/mRNA regulatory networks in GBM progression. First, to find the IGF-1-associated differentially expressed genes (DEGs) from GBM microarray data of TCGA (*n* = 528), patients were selected and subdivided into two groups, IGF-1 upregulation (with an IGF-1 z score of >0.5, *n* = 115) and IGF-1 downregulation (with an IGF-1 z score of <−0.5, *n* = 197), using the cBio Cancer Genomics Portal (cBioPortal)^16^. Hence, 5997 DEGs were selected with the false discovery rate (FDR)-adjusted *p* value < 0.05 (Supplementary Data).

**Figure 2:**
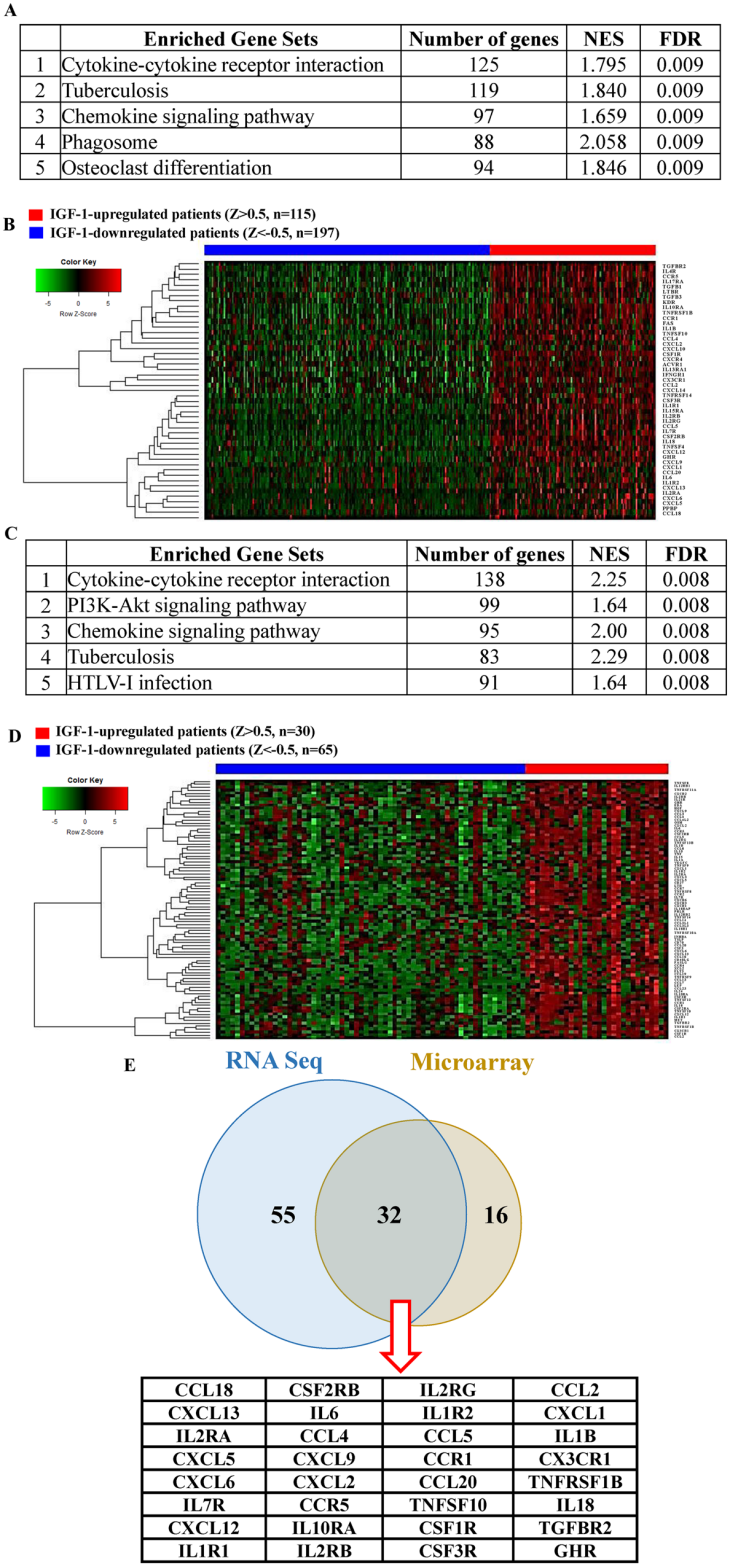
GSEA using microarray and RNA Seq data from TCGA for distinct molecular stratification of glioblastoma multiforme (GBM) patients with different insulin-like growth factor (IGF)-1 expression levels. Gene sets were based on biological processes from the Kyoto Encyclopedia of Genes and Genomes (KEGG). (**A**) The top five enriched pathways associated with the IGF-1 expression status in microarray data. The 5997 differentially expressed genes (DEGs) obtained from TCGA GBM microarray (*n* = 528) with IGF-1 Z scores of > 0.5 (IGF1-upregulated, *n* = 115) or <−0.5 (IGF1-downregulated, *n* = 197) and a false discovery rate (FDR) of <0.05 were used for the gene enrichment pathway analysis. (**B**) TCGA patients were clustered based on the supervised clustering of enriched genes in cytokine-cytokine receptor interactions. The 48 candidate genes from 125 enriched genes in cytokine-cytokine receptor interactions were used for clustering. The red and blue bars respectively mean IGF-1-upregulated and -downregulated patients. (**C**) The top five enriched pathways associated with IGF1 expression status in RNA Seq data. The 4584 DEGs obtained from TCGA GBM RNA Seq (*n* = 166) with IGF-1 Z scores of >0.5 (IGF1-upregulated, *n* = 30) or <−0.5 (IGF1-downregulated, *n* = 65) and an FDR of <0.05 were used for the gene enrichment pathway analysis. (**D**) TCGA patients were clustered based on the supervised clustering of enriched genes in cytokine-cytokine receptor interactions. The 32 candidate genes from 87 enriched genes in cytokine-cytokine receptor interactions were used for clustering. The red and blue bars respectively mean the IGF-1-upregulated and -downregulated patients. (**E**) The Venn diagram showed that 32 core enrichment genes in cytokine-cytokine receptor interactions were identified both in TCGA microarray and RNA Seq data.

To further identify enriched pathways associated with the IGF-1 expression status in GBM microarray data, we performed a Gene Set Enrichment Analysis (GSEA) with the KEGG database using TCGA microarray data for IGF-1-upregulated and -downregulated patients (Supplementary Table S1). The top five enriched pathways with an FDR of <0.01 are listed in Fig. 2A. The most significantly enriched pathway associated with the IGF-1 expression status was the cytokine-cytokine receptor interaction, which contained 48 genes in the leading edge subset (Supplementary Fig. S1A). A heatmap showed that these 48 genes with cytokine-cytokine receptor interactions were positively correlated with IGF-1-upregulated patients (Fig. 2B).

Next, to identify IGF-1-related DEGs from GBM RNA-Seq data of TCGA (*n* = 166), we repeated the same approaches mentioned above. Patients were selected and subdivided into groups of IGF-1 upregulation (with an IGF-1 z score of >0.5, *n* = 30) and IGF-1 downregulation (with an IGF-1 z score of <−0.5, *n* = 65). Hence, 4584 DEGs were determined with a FDR of <0.05 (Supplementary Data). By the GSEA, the enriched pathways with FDRs of <0.01 are listed in Fig. 2C and Supplementary Table S2. The cytokine-cytokine receptor interaction that contained 87 genes in the leading edge subset was also the most significantly enriched pathway associated with the IGF-1 expression status (Supplementary Fig. S1B, Fig. 2D). Taken together, 32 core enrichment genes were identified in both the microarray and RNA-Seq data, suggesting that genes in cytokine-cytokine receptor interactions may play vital roles in IGF-1-upregulated GBM patients.

### Investigation of the IGF-1-downregulated miRNA profile

**Figure 3:**
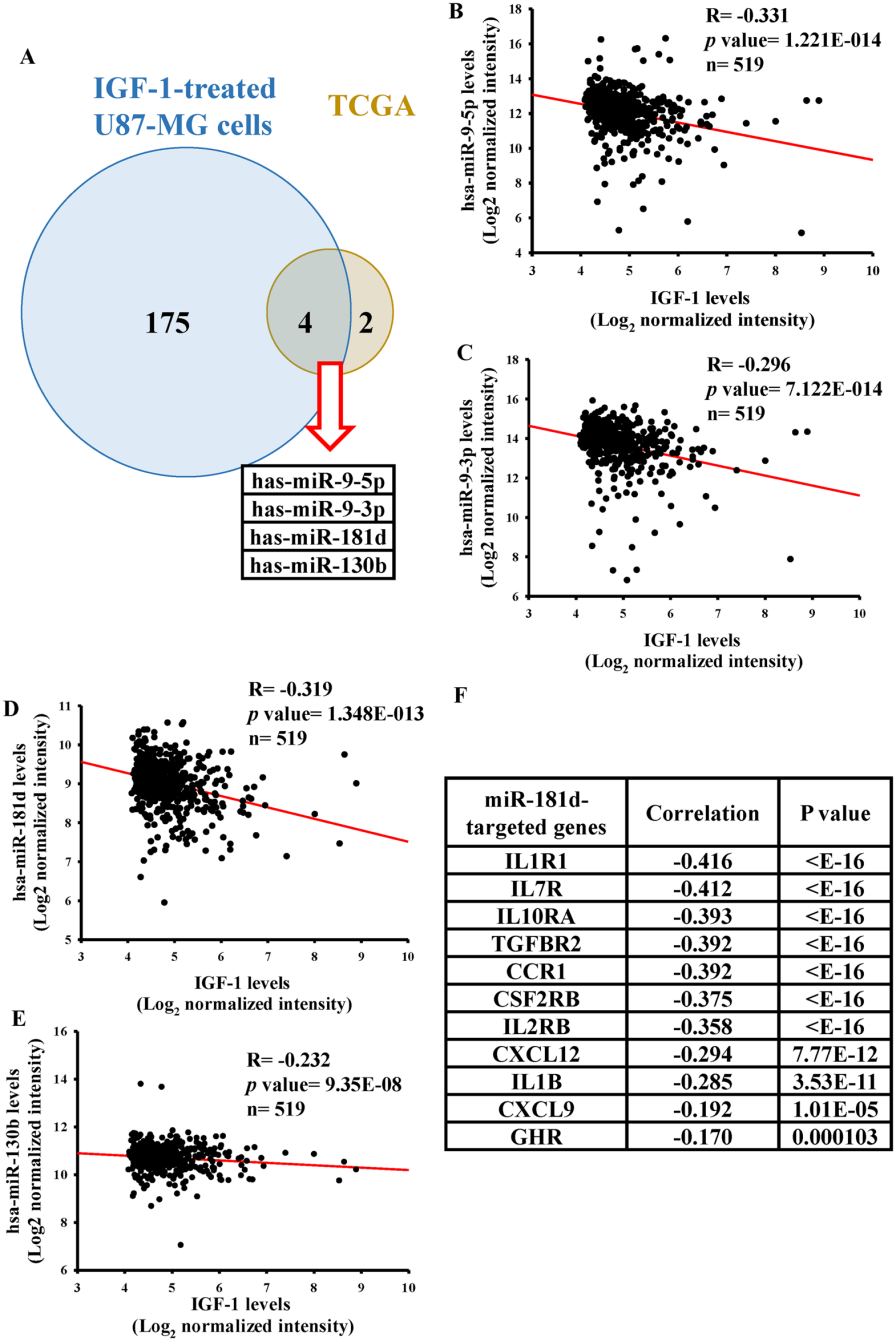
Identification of insulin-like growth factor (IGF)-1-mediated micro (mi)RNA signature by *in silico* and *in vitro* analyses. (**A**) The Venn diagram shows that four candidate miRNAs were identified in both TCGA miRNA array and IGF-1-treated U87-MG miRNA array data. Six and 179 IGF-1-downregulated miRNAs were respectively obtained from 312 TCGA miRNA arrays of GBM patients with different IGF-1 levels and the miRNA array analysis with 200 ng/ml IGF-1-treated U87 MG cells for 48 h (with a multiple of change (MC) of <0.75 and a false discovery rate (FDR) of <0.05). The four candidate miRNAs were identified in both miRNA array datasets. Expression correlations between IGF-1 and IGF-1-downregulated miRNAs included miR-9-5p (**B**), miR-9-3p (**C**), miR-181d (**D**), and miR-130b (**E**) in TCGA GBM data (*n* = 519). The correlations were measured by Spearman’s correlation analyses. (**F**) The list shows 11 putative target genes of miR-181d with an inverse correlation.

To explore the effects of miRNAs in regulating IGF-1-positively associated cytokine-cytokine receptor interaction genes, we analyzed the miRNA array data of TCGA and carried out a miRNA array analysis with IGF-1-treated U87-MG cells. The 124 differentially expressed miRNAs were found (Supplementary Table S3). Six out of 124 miRNAs were significantly downregulated in IGF-1 high patients [Fold Change (FC) of <0.75 and a FDR of <0.05]. Using a miRNA array analysis with IGF-1-stimulated U87-MG cells, 179 significantly downregulated miRNAs (with a MC of <0.75 and a FDR of <0.05) were identified (Supplementary Table S4). Finally, we found four miRNAs, i.e., miR-9-5p, miR-9-3p, miR-181d, and miR-130b, in both miRNA array data (Fig. 3A, Table 1). Furthermore, by Spearman’s correlation analyses, these four miRNAs showed significant inverse correlations with IGF-1 levels in GBM patients of TCGA (*n* = 519, Fig. 3B–E). To determine the major candidate miRNA that inhibits IGF-1-upregulated cytokine-cytokine receptor interaction genes, putative target genes were predicted by TargetScan^17^ and Spearman’s correlation analyses (Fig. 3F, Supplementary Table S5). Since miR-181d targeted the most IGF-1-upregulated cytokine-cytokine receptor interaction genes, we focused on effects of miR-181d on the IGF-1 signaling axis in further studies.

**Table 1:** Downregulated miRNAs in miRNA arrays from TCGA database and IGF-1-treated U87-MG cells.

miRNAs	TCGA database			IGF-1-treated U87-MG		
	FC	*p*. value	FDR	FC	*p*. value	FDR
hsa-miR-9-5p	0.54	<0.001	<0.001	0.24	<0.001	0.003
hsa-miR-9-3p	0.57	<0.001	<0.001	0.59	<0.001	0.004
hsa-miR-181d	0.72	<0.001	<0.001	0.62	<0.001	0.003
hsa-miR-130b	0.73	<0.001	<0.001	0.63	<0.001	0.003

FC: Fold change; FDR*:* False Discovery Rate.

### The IGF-1/miR-181d axis stratifies patient survival and is correlated with cytokine gene expressions

**Figure 4:**
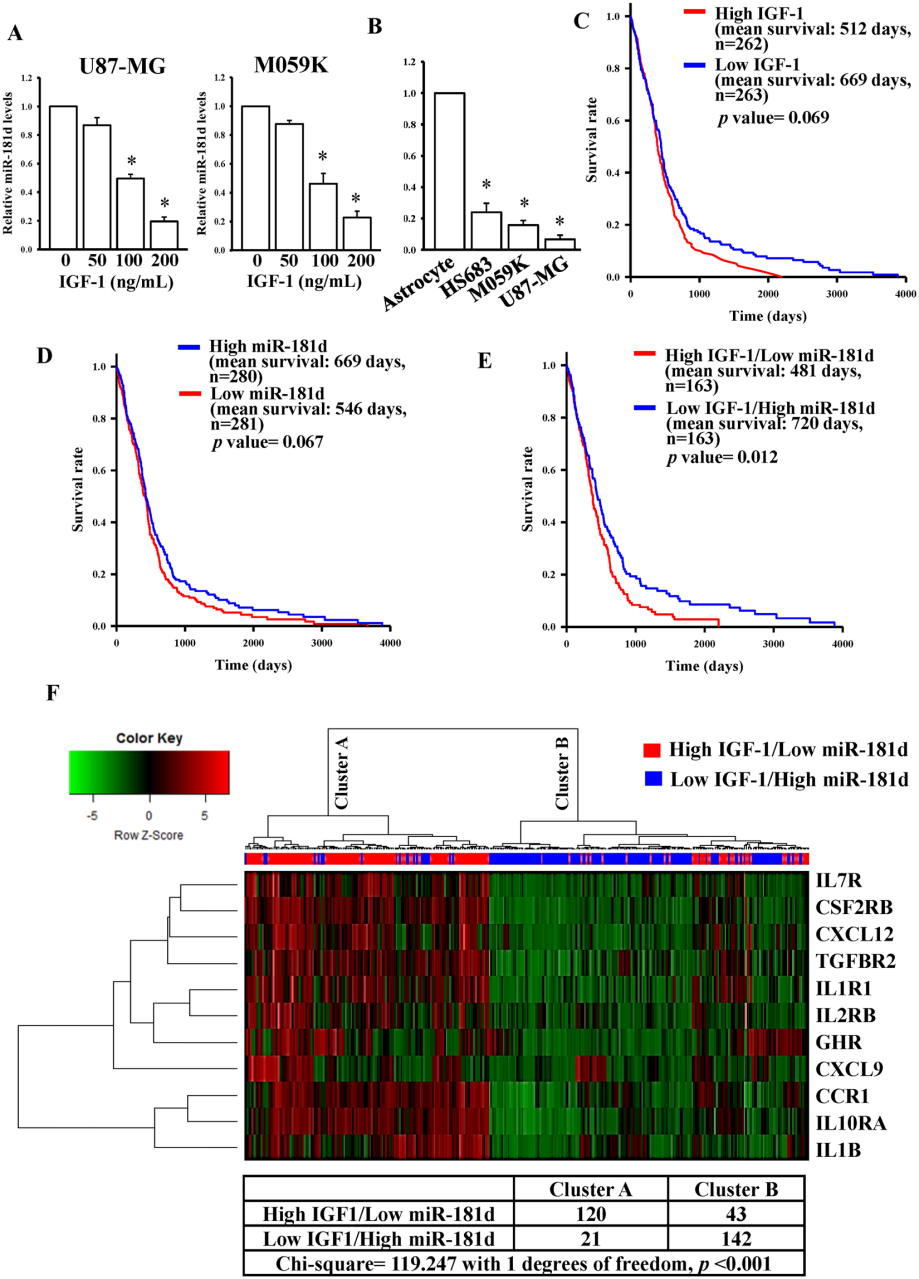
Insulin-like growth factor (IGF)-1/miR-181d status stratifies patient survival. (**A**) Dose-dependent effects of IGF-1 on miR-181d expression levels. (**B**) Detection of endogenous miR-181d levels in normal human astrocytes and three different glioma cell lines, including HS683, M059K, and U87-MG cells. After cells were treated with the indicated doses of IGF-1 for 48 h, RNA was extracted to detect miR-181d levels. Relative expression levels of miR-181d were measured with a real-time PCR. The miR-191-5p level was used as an internal control. Data are the mean ± SD of three experiments. The analyses of Kaplan-Meier survival curves in the IGF-1 (**C**), miR-181d (**D**), and IGF-1 combined with miR-181d (**E**) groups. TCGA glioblastoma multiforme (GBM) patients were divided into two groups based on the median expression cutoff points of IGF-1 and miR-181d levels. The survival rate was measured by a log-rank test. (**F**) Unsupervised hierarchical clustering of IGF1-miR-181d axis-regulated cytokine genes.

To reconfirm the array data, we measured changes in miR-181d expression levels in IGF-1-treated glioma U87-MG and M059K cells. As shown in Fig. 4A, significant and dose-dependent decreases in miR-181d levels were observed in both IGF-1-treated glioma cell lines. To compare endogenous levels of miR-181d in normal human astrocytes and three glioma cell lines, we found that lower miR-181d levels were validated in glioma cell lines than that in astrocyte cells (Fig. 4B). By survival rate analyses, the high IGF-1 patients tended to have shorter survival than low IGF-1 patients, and the low miR-181d patients tended to have shorter survival than high miR-181d patients (Fig. 4C,D). However, these were not statistically significant (mean survival day difference = 157 days and log-rank test *p* = 0.069 for the IGF-1 group; mean survival day difference = 123 days, *p* = 0.067 for the miR-181d group). Interestingly, when these two factors were combined, patients with low IGF-1 and high miR-181d demonstrated significantly longer survival than did high-IGF-1/low-miR-181d patients (Fig. 4E, mean survival day difference = 239 days, *p* = 0.012), suggesting that the combined IGF-1/miR-181d signature could be a significant prognostic indicator, as opposed to IGF-1 or miR-181d alone. This is a nice finding. By further using the Cox proportional hazards model with clinical variables for GBM patients of TCGA cohort, the high-IGF-1/low-miR-181d status, G-CIMP phenotype, age, and Karnofsky performance score were independently correlated with overall survival, while gender was not associated with overall survival (hazard ratio (HR) = 1.435, *p* = 0.022) (Table 2). In addition, we also calculated correlations between overall survival and three other miRNAs with a high-IGF-1 status, and high-IGF-1/low-miR-9-3p status was associated with overall survival (HR = 1.456, *p* = 0.026) (Supplementary Table S6). As a consequence, the results indicated that high IGF-1/low miR-181d is an independent prognostic factor for GBM.

**Table 2:** Statistical calculations for high IGF-1/low miR-181 status.

Covariate	Hazard Ratio	95% Conf-U	95% Conf-L	stdErr	*p*. value
High IGF1/Low miR-181d	1.435	1.956	1.053	0.158	0.022
G-CIMP	3.876	7.547	1.991	0.34	<0.001
Age > 60	1.433	1.942	1.058	0.155	0.02
Karnofsky score ≥ 80	1.988	2.807	1.408	0.176	<0.001
Gender (male)	1.061	1.446	0.778	0.158	0.708

IGF1-miR-181 status is a strong and independently significant prognostic factor among Karnofsky performance status, age, gender and CIMP status. stdErr: stander error; Conf-L/U: Confidence interval lower limit/upper limit.

To further verify relationships between the IGF-1/miR-181d axis and miR-181d-targeted cytokine genes, we subdivided these 11 miR-181d-targeted cytokine genes into cluster A and cluster B groups by unsupervised hierarchical clustering (Fig. 4F). We found that 85% of patients in cluster A had a high-IGF-1/low-miR-181d status, whereas 77% of patients in cluster B had a low-IGF-1/high-miR-181d status. By conducting a Chi*-*squared test, the IGF-1 miR-181d status was shown to be highly associated with cytokine gene expression patterns (Chi-squared = 119.2 with 1 degree of freedom, *p* ≤ 0.001).

### Identification of IL-1b and CCR1 as direct target genes of miR-181d

**Figure 5:**
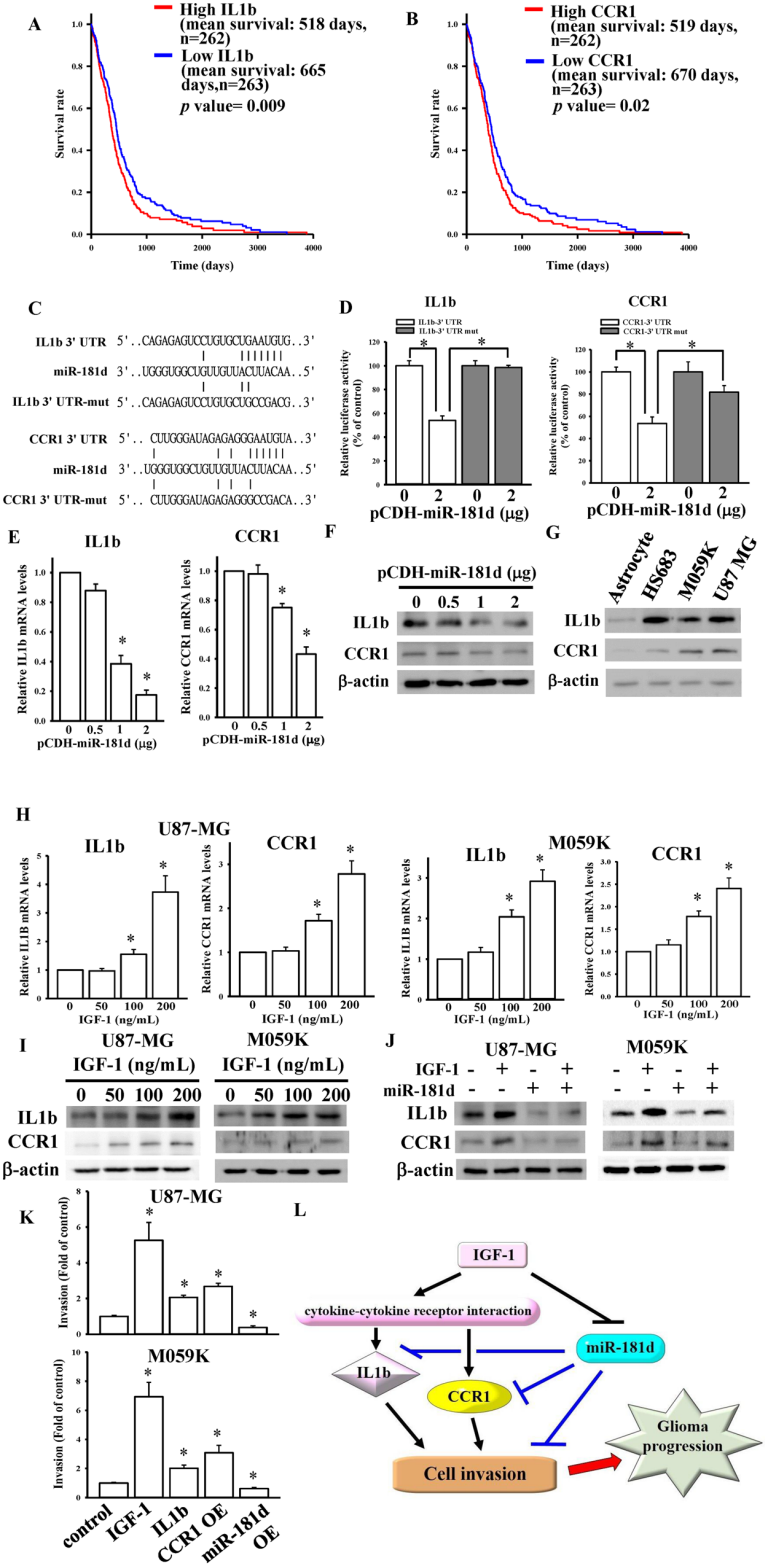
Interleukin (IL)-1b and C-C chemokine receptor type 1 (CCR1) were identified as direct target genes of miR-181d. Kaplan-Meier survival curves were analyzed in the IL-1b (**A**) and CCR1 (**B**) groups. The survival rate was calculated by multivariate permutation tests (with a false discovery rate (FDR) of <0.01). TCGA GBM patients were divided into two groups based on the median expression cutoff points of IL-1b and CCR1 levels. (**C**) Schematic diagram of potential miR-181d-targeted sites in the 3′-untranslated region (UTR) of the *IL-1b* and *CCR1* genes. (**D**) Effects of miR-181d on 3′UTR luciferase activity of the *IL-1b* and *CCR1* genes. To test for miR-181d’s effect, different doses of miR-181d-expressing plasmids were co-transfected with 500 ng of the pmiRGlo-3′UTR or mutant 3′UTR of the *IL-1b* and *CCR1* genes. Luciferase activity was measured in these cells 24 h after transfection. Effects of miR-181d overexpression on mRNA (**E**) and protein (**F**) expressions of the *IL-1b* and *CCR1* genes. (**G**) Detection of endogenous protein levels of the *IL-1b* and *CCR1* genes in normal human astrocytes and three different glioma cell lines including HS683, M059K, and U87-MG cells. Dose-dependent effects of IGF-1 on mRNA (**H**) and protein (**I**) expressions of the *IL-1b* and *CCR1* genes. Data are the mean ± SD of three experiments. *p < 0.05. (**J**) The effects of miR-181d on IGF-1-mediated IL-1b and CCR1 protein levels. (**K**) The effects of IGF1-miR181d-cytokines axis on glioma cell invasion. After cells were respectively treated with 100 ng/ml IGF-1, 10ng/ml IL1b, or transfecting with CCR1 and miR-181d overexpressing plasmids, the cell invasion changes were measured by matrigel invasion assays. Data are the mean ± SD of three different fields. *p < 0.05 compared with control. (**L**) Schematic diagram shows IGF-1 expressions are highly associated with cytokine-cytokine receptor interaction, especially in regulations of IL1b and CCR1, both are miR-181d direct target genes. IGF-1 enhances IL1b and CCR1 expressions via reducing miR-181d levels, resulting in glioma cell invasion and progression.

To determine which IGF-1-mediated cytokine-cytokine receptor interaction genes targeted by miR-181d were associated with overall survival, univariate proportional hazard models were conducted. We found that only the IL-1b and CCR1 genes were significantly correlated with overall survival (*p* values < 0.01) (Table 3, Supplementary Table S7). To further validate this by a log-rank test (Fig. 5A,B), high-IL-1b or high-CCR1 patients had shorter survival than low-IL-1b or low-CCR1 patients (mean survival day difference = 147 days and log-rank test *p* = 0.009 for the IL-1b group; mean survival day difference = 151 days, *p* = 0.02 for the CCR1 group). By a TargetScan analysis, we found that both *IL-1b* and *CCR1* were putative target genes of miR-181d (Fig. 5C). To further confirm that *IL-1b* and *CCR1* are miR-181d target genes, 3′UTRs of both genes containing a miR-181d-binding site were respectively cloned into the pmiRGlo-reporter plasmid to conduct 3′UTR reporter assays. As shown in Fig. 5D, 2 µg of miR-181d-overexpressing plasmids (Supplementary Fig. S2) significantly decreased the luciferase activities of IL-1b and CCR1. To further validate that IL-1b and CCR1 levels could be regulated by miR-181d via 3′UTR binding, five nucleotides located in the critical binding region of the 3′UTRs of both genes were mutated by site-directed mutagenesis (Fig. 5C). As shown in Fig. 5D, miR-181d had no effect on luciferase activity after mutating the miR-181d-targeted site. We also directly tested the effect of miR-181d on expressions of both genes and found that transient transfection of miR-181d into U87-MG cells significantly and dose-dependently decreased mRNA and protein levels of IL-1b and CCR1 as measured by real-time qPCR assays (Fig. 5E) and an immunoblotting analysis (Fig. 5F), suggesting that both IL-1b and CCR1 are direct target genes of miR-181d.

**Table 3:** Survival analysis of miR-181d-targeted genes.

Gene	Hazard Ratio	SD of log intensities	*p* value
IL1B	1.129	1.203	0.0016
CCR1	1.165	0.981	0.0019

SD of log intensities: standard deviation of the log 2 of the gene expression level.

### Effects of miR-181d on IGF-mediated IL-1b and CCR1 levels

Since previous results showed that higher IL-1b and CCR1 levels were found in GBM array data of TCGA, we also measured endogenous protein levels of IL-1b and CCR1 in astrocyte and glioma cell lines. As shown in Fig. 5G, IL-1b and CCR1 protein levels were higher in glioma cells than that in astrocyte cells. To test the effects of IGF-1 on endogenous levels of IL-1b and CCR1 in glioma cells, we measured mRNA and protein levels of IL-1b and CCR1 in IGF-1-stimulated glioma cells by real-time qPCR assays and immunoblotting analyses. IGF-1 induced dose-dependent increases in both mRNA and protein levels of IL-1b and CCR1 (Fig. 5H,I). To further identify if miR-181d is really involved in IGF-1-upregulated IL-1b and CCR1 levels, we measured protein levels of IL-1b and CCR1 after transfection of miR-181d-overexpressing plasmids combined with IGF-1 treatment. As shown in Fig. 5J, miR-181d overexpression significantly attenuated IGF-1-upregulated IL-1b and CCR1 levels. Finally, we tried to investigate the effects of IGF1-miR181d-cytokines axis on glioma cell growth and invasion. First, we tested the effect of IGF-1 stimulation on U87-MG cell growth by MTT assays. Surprisingly, IGF-1 stimulations did not show significant increase in cell growth (Supplementary Figure S3A). Next, we tested the effect of IGF-1 stimulation on U87-MG cell invasion by matrigel invasion assays. As shown in Fig. 5K, Supplementary Figure S3B and C, IGF-1 stimulations significantly enhanced both U87-MG and M059k cell invasion. Furthermore, by treating with IL1b recombinant proteins, or respectively transfecting with CCR1 (Supplementary Figure S3D) and miR-181d genes, we found that both IL-1b and CCR1 could obviously induce glioma cell invasion. In contrast, overexpression of miR-181d significantly reduced glioma cell invasion. Taken together, the IGF-1-mediated miR-181d/cytokine gene regulatory network may involve in regulating cell invasion of GBM progression.

## Discussion

Cytokines, important communicators in the tumor microenvironment, possess either pro- or anti-inflammatory activity, and immunosuppressive activity, and participate in GBM progression and aggressiveness^18^. Increasing evidence suggests that both IGF-1 signaling and miRNA regulation are highly associated with cytokine gene expressions in disease development^7, 19^. It is necessary to clarify relationships among IGF-1, cytokines, and miRNAs in the GBM development process. In the present study, we found that 32 core enrichment genes of cytokine-cytokine receptor interactions demonstrated positive associations with IGF-1 levels in GBM patients by analyzing the microarray and RNA Seq data from TCGA. We also identified four IGF-1-downregulated miRNAs in miRNA array data from TCGA and IGF-1-treated U87-MG cells. By an integrative miRNA/mRNA regulatory network analysis, 11 IGF-1-upregulated cytokine-cytokine receptor interaction genes were targeted and showed inverse correlations with miR-181d. Furthermore, we suggest that the IGF-1/miR-181d axis, IL-1b, and CCR1 levels were highly associated with patient survival. Finally, reduced endogenous miR-181d and increased IL-1b/CCR1 levels were identified in IGF-1-stimulated glioma cells. Overexpression of miR-181d significantly attenuated IGF-1-upregulated IL-1b and CCR1 levels, which were recognized as direct target genes of miR-181d. Consequently, IGF-1-enhanced cytokine IL-1b and CCR1 signaling via inhibiting miR-181d is involved in glioma progression (Fig. 5L).

Several studies reported the relationships between IGF-1 signaling and miRNA regulation involved in mediating physiological processes and disease development, especially in glioma. The miR-7^20^, miR-181b^21^, miR-139^22^, and miR-323-5p^23^ have been reported to target to the IGF-1 receptor, resulting in influencing the effects of IGF-1 signaling on glioma development. In our previous study, we also found that miR-128 could target to IGF-1-mediated mammalian target of rapamycin (mTOR) signaling in temozolomide-induced glioma cell apoptotic death, suggesting that microRNAs play critical roles in regulating IGF-1 signaling involved in glioma progression. In the present study, we found that miR-181d levels were significantly reduced in GBM cells upon IGF-1 treatment. A significant inverse correlation between miR-181d and IGF-1 levels was observed in GBM patients of TCGA. Similarly, according to Guo *et al*.’s miRNA array study published in GEO datasets (GSE57657), miR-181d levels were also lower in IGF-1-treated human metaphase I oocytes, suggesting that miR-181d gene expression can be inhibited by IGF-1 stimulation. Furthermore, high-IGF-1/low-miR-181d patients showed significantly shorter survival than low-IGF-1/high-miR-181d patients, and thus they could be an independent prognostic factor in GBM. However, further study is required to understand the molecular mechanisms of IGF-1-reduced miR-181d expression.

Multiple functions of miR-181d were reported in the regulation of both normal physiological processes and tumorigenesis. In gliomas, by targeting K-ras, Bcl-2, miR-181d was recognized as a glioma suppressor^13^. Furthermore, miR-181d directly targeted and inhibited methyl-guanine-methyl-transferase (MGMT) expression in glioma cells^14^. miR-181d could be used as a predictive biomarker for TMZ response in gliomas. However, most functions of miR-181d in glioma progression are still unclear. In the present study, we found inverse correlations between miR-181d and the cytokines, IL-1b and CCR1, in GBM patients. We also demonstrated that both IL-1b and CCR1, directly targeted by miR-181d, were involved in IGF-1 signaling and were highly associated with patient survival. Taken together, miR-181d plays a crucial vital role in mediating the crosstalk between IGF-1 signaling and cytokine expressions in glioma progression.

CCR1 (CD191), a chemokine receptor, is expressed by several types of white blood cells, lymphocytes, astrocytes, neuron, and tumor cells^24^. CCR1 can bind to several chemokines including CCL2, CCL5, and CCL8. Upon ligand binding, CCR1 directly or indirectly stimulates tumor growth, cell adhesion, and cytokine secretion. For example, CCL5 promotes murine microglia migration through binding to CCR1^25^. The role of CCR1 has been characterized for several inflammatory conditions and tumorigenesis. However, little is known about the roles of CCR1 in glioma progression. IL-1b, one member of the IL-1 family, is a proinflammatory cytokine in cancer pathogenesis^26^. IL-1b stimulation promotes U87-MG cell viability and proliferation^27^. Aberrant expression of IL-1b by GBM cells influenced the migratory capacity, unique gene signature, and proinflammatory signaling^28, 29^. Furthermore, combined IL-1b and TGF-β treatment induced neurosphere formation and increased tumorigenicity of glioma cells^30^. All these findings suggest that IL-1b-mediated neuroinflammatory cascades contribute to glioma progression. However, until now, no studies have reported that expression of both of these genes can be regulated by miRNAs in glioma progression.

In the present study, by an integrative network analysis, we identified relationships between the IGF-1/miR-181d axis and cytokine genes in glioma progression. We also found that all of these factors were significantly associated with GBM patient survival. Finally, we validated that CCR1 and IL-1b are direct target genes of miR-181d and are involved in IGF-1 signaling. Nevertheless, some limitations still exist in our study. Compared to 714 human miRNAs in our array data, only 470 miRNAs were validated in TCGA miRNA array data. We speculated that some important and unknown functional miRNAs were ignored in our present analyses. The tumorigenic functions of CCR1 and IL-1b in the IGF-1/miR-181d axis need to be further studied. As a consequence, we concluded that IGF-1-enhanced cytokine expressions targeted by miR-181d in glioblastoma could be beneficial to understanding glioma development and to provide novel therapeutic targets of future glioma therapies.

## Materials and Methods

### Chemicals and reagents

Human glioblastoma Hs-683, M059K, and U87-MG cells were purchased from the Bioresource Collection and Research Center (Hsinchu City, Taiwan). Primary human astrocytes were purchased from Thermo Fisher Scientific (Waltham, MA, USA). Other cell culture-related reagents were purchased from GIBCO-BRL (Grand Island, NY, USA). Anti-IL-1b, CCR1, and β-actin antibodies were purchased from GeneTex (Hsinchu City, Taiwan). Polyvinylidene difluoride (PVDF) membranes, and the enhanced chemiluminescence (ECL) solution (cat. no. WBKLS0500) were purchased from Millipore (Billerica, MA, USA). The human IGF-1 recombinant protein (cat. no. PHG0071), Trizol® reagent (cat. no. 15596026), Lipofectamine 3000 (cat. no. L3000015), and secondary antibodies were purchased from Invitrogen (Thermo Fisher Scientific). SYBR® Green PCR (polymerase chain reaction) Master Mix (cat. no. 4309155), the MultiScribe (tm) Reverse Transcriptase Kit (cat. no. N8080234), TaqMan Advanced miRNA cDNA Synthesis Kit (cat. no. A28007), TaqMan® Advanced miR-181d (cat. no. 479517_mir), and TaqMan® Advanced miR-191-5p (cat. no. 477952_mir) were purchased from Applied Biosystems (Thermo Fisher Scientific). The dual-luciferase reporter assay system (cat. no. E1910) was purchased from Promega (Madison, WI, USA). Matrigel matrix (cat. no. 354234) was purchased from Corning (Corning, NY, USA). IL-1b recombinant proteins (cat. no. 0103B95R1) was purchased from PeproTech (Rocky Hill, NJ, USA). CCR1 gene plasmid was purchased from Genscript (Piscataway, NJ, USA). Primer sets were synthesized by Genomics BioSci & Tech (Xizhi, New Taipei City, Taiwan). Unless otherwise specified, all other reagents were of analytical grade.

### Selection of patient data from TCGA

GBM patients were selected based on mRNA expression levels of IGF-1. Using the cBioPortal web (www.cbioportal.org)^16^, patients with a z score of >0.5 (IGF-1 upregulation) or <−0.5 (IGF-1 downregulation) compared to the overall distribution were selected. The level 3 microarray, and RNA Seq and miRNA array data were respectively downloaded from TCGA website (https://tcga-data.nci.nih.gov/tcga/).

### Analysis of the microarray, miRNA array, and RNA sequencing profile from GBM patients in TCGA

Since the downloaded mRNA and miRNA array data had already been normalized, differentially expressed genes (DEGs) and miRNAs between IGF-1 upregulated and downregulated patients were analyzed using the Limma package^31^. Significant genes and miRNAs with a false discovery rate (FDR)-adjusted *p* value threshold of <0.05 were further analyzed. For RNA Seq data, the value of RNA Seq by Expectation Maximization (RSEM) was provided. In order to identify downstream DEGs, the RSEM value was transformed into (log2(RSEM +1)), where +1 was added to avoid excessive variations due to very small values. Log transformation was performed to avoid overrepresentation of extreme values. The gene profile was compared between patients with a z score of >0.5 and of <−0.5. DEGs were also identified using the Limma package with an FDR-adjusted *p* value threshold of 0.05.

### Gene set enrichment analysis (GSEA)

In order to access the potential pathway related to IGF-1 in GBM patients, the clusterProfiler package^32^ was used to perform a GSEA with the Kyoto Encyclopedia of Genes and Genomes (KEGG). By applying the GSEA algorithm with DEGs between IGF-1-upregulated and -downregulated patients, the most significant pathways with a *p* value of <0.01 were found. The leading edge subsets of cytokine and cytokine receptor interactions found by GSEA were plotted in a heatmap using the Gplots package.

### miRNA array analysis and target gene prediction

Total RNA was respectively isolated from U87-MG cells with or without 200 ng/ml IGF-1 treatment for 48 h. miRNA expression profiling was performed using the Human microRNA OneArray^®^ vers. 6.2 (Phalanx Biotech Group, Hsinchu, Taiwan). All experiments, including complementary (c)RNA amplification, hybridization, image scanning with an Axon 4000 scanner (Molecular Devices, Sunnyvale, CA, USA), and statistical analysis with Genepix software (Molecular Devices), were conducted by the Phalanx Biotech Group (Hsinchu, Taiwan). The log2 (ratio) was calculated by the pair-wise combination and error-weighted average. Adjusted (adj.) *p* values were calculated with the Benjamini*-*Hochberg multiple testing correction. Significant differentially expressed miRNA lists filtered for adj. *p* values (differentially expressed) of <0.05 and a log2 (ratio) of ≦−0.415 (0.75 multiples of change) cutoff were applied for further analysis. All target genes of differential miRNAs were predicted using TargetScan^17^. Significant correlations between miRNAs and their target genes from TCGA array data (*n* = 519) were calculated using a Spearman correlation, and genes with different and significantly negative correlations were filtered out. The functional annotation of these candidate genes was performed with KEGG.

### Unsupervised hierarchical clustering and survival analysis

Putative IGF-1-miR-181d axis-regulated cytokine and cytokine receptor interaction genes were selected and hierarchically clustered in high-IGF-1/low-miR-181d and low-IGF-1/high-miR-181d patients. Sample dissimilarity was calculated using the Spearman rank, the dissimilarity matrix was clustered using the Ward-linkage, and heatmaps were generated using the Gplots package. Associations between clusters and the IGF-1-miR-181d status were calculated via a Chi-squared test. For the survival analysis, patients were categorized into different groups based on median expression cutoff points of IGF-1 and miR-181d. Differences in overall survival between these groups were investigated using a log-rank test, and results were plotted as a Kaplan-Meier curve. For high-IGF-1/low-miR-181d and low-IGF-1/high-miR-181d patients, Cox proportional hazards models were calculated considering the patient’s age, gender, Karnofsky performance status, and glioblastoma CpG island methylator phenotype (G-CIMP). The IGF1-miR-181d axis-regulated genes in the cytokine and cytokine receptor interaction pathway were selected to perform a univariate Cox’s proportional-hazards regression to identify genes for which expression was correlated with survival.

### Cell culture, treatments, and transfection

U87-MG cells were maintained in minimum essential Eagle’s medium (MEM); M059K cells were maintained in a 1:1 mixture of Dulbecco’s modified Eagle’s medium (DMEM) and Ham’s F12 medium with 2.5 mM L-glutamine; HS-683 cells were maintained in DMEM with 4 mM L-glutamine; and astrocyte cells were maintained in DMEM with N-2 Supplement. All cells were supplemented with 10% fetal bovine serum (Biological Industries, Cromwell, CT, USA), 100 units/ml penicillin, 100 μg/ml streptomycin, 1 mM sodium pyruvate, and 1 mM nonessential amino acids at 37 °C in a 5% CO_2_ incubator. For IGF-1 treatment, indicated doses of IGF-1 were added to overnight-cultured cells for 48 h. To conduct the transfection experiments, cells were seeded in a 12-well plate at a density of 10^5^ cells/well. After achieving 70% confluence in a well, indicated doses of pCDH-miR-181d, an empty pCDH vector, and 500 ng pmiRGLO 3′UTR reporter plasmids were respectively transfected with Lipofectamine 3000 (Invitrogen) according to the manufacturer’s instructions. After 24 h of incubation, cells were lysed for further study.

### Immunoblot analysis

Cells were harvested in RIPA buffer (1% Nonidet P-40, 0.5% deoxycholate, and 0.1% sodium dodecylsulfate (SDS) in phosphate-buffered saline (PBS)) containing a protease inhibitor cocktail (Calbiochem, Billerica, MA, USA) and centrifuged at 12,000 rpm for 10 min at 4 °C. The supernatant was used as the total cell lysate. Lysates (20 μg) were denatured in 2% SDS, 10 mM dithiothreitol, 60 mM Tris-hydrochloric acid (Tris-HCl, pH 6.8), and 0.1% bromophenol blue, and loaded onto 10% and 15% polyacrylamide/SDS gels. The 10% and 15% polyacrylamide/SDS gels were used to analyze target proteins whose respective molecular weights were >50 and <50 kDa. Separated proteins were then transferred onto a polyvinylidene difluoride membrane. The membrane was blocked for 1 h at room temperature in PBS containing 5% nonfat dry milk and incubated overnight at 4 °C in PBS-T containing the primary antibody. The membrane was washed in PBS-T, incubated with the secondary antibody conjugated to horseradish peroxidase for 1 h at room temperature, and then washed in PBS-T. The primary and secondary antibodies were respectively diluted to 1:1000 and 1:3000 with PBS-T buffer. An enhanced chemiluminescence (ECL) non-radioactive detection system was used to detect the antibody-protein complexes.

### RNA isolation and quantitative real-time reverse-transcription polymerase chain reaction (RT-qPCR)

Total RNA from cultured cells was extracted using Trizol® according to the manufacturer’s instructions. RNA quality was checked using A260/A280 readings. Complimentary (c)DNA was synthesized from 1 μg of total RNA using a random primer and the MultiScribe (tm) Reverse Transcriptase Kit. cDNA was diluted 1:30 with PCR-grade water and then stored at −20 °C. To detect miRNA levels, cDNA was synthesized with a TaqMan® Advanced miRNA cDNA Synthesis Kit (Applied Biosystems). TaqMan® Advanced miRNA assays were used to detect miR-181d levels. To quantify miRNA expression levels, miR-191-5p was used as an internal control. Specific primers for human IL-1b, CCR1, and GAPDH for the real-time qPCR are listed in Supplementary Table S8. Gene expression levels were quantified with the Applied Biosystems StepOnePlus™ System (Thermo Fisher Scientific) with pre-optimized conditions. Each PCR was performed in triplicate using 5 μl of 2x SYBR Green PCR Master Mix, 0.2 μl of primer sets, 1 μl cDNA, and 3.6 μl of nucleotide-free H_2_O to yield 10 μl per reaction. Expression rates were calculated as the normalized C_T_ difference between the control and sample after adjusting for the amplification efficiency relative to the expression level of GAPDH.

### Construction of full-length miR-181d-overexpressing plasmids

To construct miR-181d-overexpressing plasmids, the 300-bp length of the miR-181d gene was generated by PCR amplification using primers listed in Supplementary Table S8. The following thermal profile was used for the PCR amplification of 500 ng cDNA on a GeneAmp PCR system 9700 (Applied Biosystems): an initial denaturation step at 95 °C for 5 min, followed by 40 cycles of 94 °C for 1 min, 58 °C for 1 min, and 72 °C for 1 min, with a final extension at 72 °C for 10 min. PCR products were analyzed by agarose gel electrophoresis. All PCR products were cloned into pGEM-T Easy (Promega, Madison, WI, USA) and sequenced. After *Bam*HI/*Eco*RI digestion, miR-181d cDNA was cloned into pCDH vectors (System Biosciences, Palo Alto, CA, USA) to form a construct called pCDH-miR-181d.

### Construction of the IL-1b and CCR1 3′UTR reporter plasmid and mutagenesis

The PCR was performed using sets of primers specific for IL-1b and the CCR1 3′UTR, of which the forward primer was XhoI-site-linked and the reverse primer was XbaI-site-linked. PCR primers are listed in Supplementary Table S8. U87 MG genomic DNA was used as a template. The PCR product was digested with XhoI and XbaI, and cloned downstream of the luciferase gene in the pMIRGLO-REPORT luciferase vector (Promega). These vectors were sequenced and respectively named pMIRGLO-IL-1b-3′UTR and pMIRGLO-CCR1-3′UTR. Site-directed mutagenesis of the miR-181d target site in IL-1b and CCR1 3′UTR was carried out using the QuikChange Site-Directed Mutagenesis Kit (Stratagene), and these vectors were respectively named pMIRGLO-IL-1b-3′U-MUT and pMIRGLO-CCR1-3′U-MUT. For the reporter assays, cells were transiently transfected with the wild-type or mutant reporter plasmids, and the miR-181d plasmids using Lipofectamine 3000 (Invitrogen). The reporter assay was performed at 24 h post-transfection using the Luciferase Assay System (Promega). The dual Renilla luciferase value was used as an internal control.

### Matrigel invasion assays

Matrigel invasion assays were conducted using transwell inserts (25 mm polycarbonate membrane, 8 μm pore size; Millipore) coated with Matrigel matrix (Corning, NY, USA). After cells were respectively treated with 100 ng/ml IGF-1, 10 ng/ml IL1b, or transfecting with CCR1 and miR-181d overexpressing plasmids, 5 × 104 cells were placed in the top chamber of transwell migration chambers. After 48 hours, Cells that had not migrated to the lower chamber were removed from the upper surface of the transwell membrane with a cotton swab. Migrating cells on the lower membrane surface were fixed in 100% methanol (−20 °C, 15 min), stained, photographed and counted with a microscope at 100x magnification. Experiments were assayed in triplicate, and at least five fields were counted in each experiment. Statistical analysis was carried out using Student’s t-test (paired).

### Statistical analysis

All data are presented as the mean ± standard deviation (SD). Significant differences among groups were determined using an unpaired Student’s *t*-test. A value of *p* < 0.05 was taken as an indication of statistical significance. All figures shown in this article were obtained from at least three independent experiments with similar results.

## Electronic supplementary material

Dataset 1

Supplementary figure and Table
