# Supplementary material for: Identification of IGF-1-enhanced cytokine expressions targeted by miR-181d in glioblastomas via an integrative miRNA/mRNA regulatory network analysis
Source: Sci Rep. 2017 Apr 7;7:732. doi: 10.1038/s41598-017-00826-0 (PMC5429683; doi:10.1038/s41598-017-00826-0)

Identification of IGF-1-enhanced cytokine expressions targeted by miR-181d in glioblastomas via an integrative miRNA/mRNA regulatory network analysis

Kuo-Hao Ho[1*], Peng-Hsu Chen[2*], Edward Hsi[3], Chwen-Ming Shih[2,6], Wei-Chiao Chang[4,5], Chia-Hsiung Cheng[2,6], Cheng-Wei Lin[2,6], Ku-Chung Chen[2,6#]

1. Department of Clinical Pharmacy, School of Pharmacy, Taipei Medical University, Taipei, Taiwan 2. Graduate Institute of Medical Sciences, College of Medicine, Taipei Medical University, Taipei, Taiwan 3. Graduate Institute of Biomedical Sciences, China Medical University, Taichung, Taiwan 4. Department of Clinical Pharmacy, Master Program for Clinical Pharmacogenomics and Pharmacoproteomics, School of Pharmacy, Taipei Medical University, Taipei, Taiwan 5. Department of Pharmacy, Taipei Medical University-Wanfang Hospital, Taipei, Taiwan 6. Department of Biochemistry and Molecular Cell Biology, School of Medicine, College of Medicine, Taipei Medical University, Taipei, Taiwan

*These authors contributed equally to the work.

[#] Corresponding authors: Dr. Ku-Chung Chen

Supplementary Figure legends

Supplementary Figure S1. GSEAs with insulin-like growth factor (IGF)-1-associated differentially expressed genes (DEGs). GSEA diagrams showing that the gene set related to cytokine-cytokine receptor interactions was enriched in IGF-1-upregulated patients from TCGA microarray (A) and RNA Seq (B) data. The 48 and 32 core enrichment genes in (A) and (B) were identified in the leading edge subset.

Supplementary Figure S2. Endogenous levels of miR-181d in miR-181d-overexpressing cells. After cells were transfected with the indicated doses of miR-181d-expressing plasmids or empty pCDH vectors for 24 h, relative levels of miR-181d were measured with a real-time PCR. The miR-191-5p level was used as an internal control. Data are the mean ± SD of three experiments.

Supplementary Figure S3. The effects of IGF-1-miR181d-cytokines axis on glioma cell growth and invasion. (A) The effect of IGF-1 on U87-MG cell growth. After cells were treated with 100 ng/ml IGF-1 for 24 h, the cell growth was measured by MTT assays. (B and C) The effects of IGF-1, IL1b, CCR1, and miR-181d on U87-MG and M059K cell invasion. After cells were respectively treated with 100 ng/ml IGF-1, 10ng/ml IL1b, or transfecting with CCR1 and miR-181d overexpressing plasmids, the cell invasion changes were measured by matrigel invasion assays. (D) Overexpression of CCR1 genes. After cells were transfected with 1μg pcDNA3-CCR1 plasmids, the CCR1 protein levels were measured by immunoblotting assays.

Supplementary Table S1. Gene set enrichment analysis of IGF-1-associated DEGs in microarray

Enrichment gene set	Number of genes	NES	*p*. value	FDR
Cytokine-cytokine receptor interaction	125	1.795177	0.001001	0.008919
Tuberculosis	119	1.839826	0.001001	0.008919
Chemokine signaling pathway	97	1.65872	0.001002	0.008919
Phagosome	88	2.058306	0.001003	0.008919
Osteoclast differentiation	94	1.846426	0.001003	0.008919
Herpes simplex infection	98	1.783689	0.001003	0.008919
Influenza A	104	1.742874	0.001005	0.008919
Toll-like receptor signaling pathway	72	1.659677	0.001006	0.008919
TNF signaling pathway	71	1.666449	0.001006	0.008919
Toxoplasmosis	76	1.756359	0.001007	0.008919
Lysosome	75	1.69355	0.001008	0.008919
Cell adhesion molecules (CAMs)	76	1.941922	0.001009	0.008919
Platelet activation	69	1.758676	0.001013	0.008919
Systemic lupus erythematosus	61	2.231625	0.001013	0.008919
NF-kappa B signaling pathway	60	1.810673	0.001015	0.008919
Fc gamma R-mediated phagocytosis	49	1.805769	0.001016	0.008919
Leukocyte transendothelial migration	61	1.797207	0.001016	0.008919
Leishmaniasis	58	2.116371	0.001017	0.008919
Amoebiasis	56	1.760917	0.001017	0.008919
Rheumatoid arthritis	58	2.068267	0.001017	0.008919
Complement and coagulation cascades	51	2.16162	0.001018	0.008919
Antigen processing and presentation	39	2.093406	0.001025	0.008919
Pertussis	52	1.881533	0.001026	0.008919
Inflammatory bowel disease (IBD)	44	2.09334	0.001026	0.008919
Hematopoietic cell lineage	48	2.12388	0.001027	0.008919
Viral myocarditis	40	1.98547	0.001029	0.008919
Legionellosis	37	1.844959	0.001034	0.008919
Staphylococcus aureus infection	40	2.442836	0.001035	0.008919
Protein digestion and absorption	36	1.750502	0.001042	0.008919
Type I diabetes mellitus	31	1.930238	0.001043	0.008919
Malaria	35	1.96528	0.001053	0.008919
Autoimmune thyroid disease	31	1.981315	0.001054	0.008919
Allograft rejection	28	1.969347	0.001055	0.008919
Graft-versus-host disease	28	2.040211	0.001056	0.008919
Intestinal immune network for IgA production	28	2.105781	0.001081	0.008919

Asthma	20	2.22251	0.001092	0.008919
Primary immunodeficiency	22	1.757746	0.001107	0.008919
Proteoglycans in cancer	119	1.525951	0.002002	0.014879
Measles	81	1.553189	0.002016	0.014879
Natural killer cell mediated cytotoxicity	65	1.66563	0.002026	0.014879
Salmonella infection	48	1.656164	0.002047	0.014879
Arachidonic acid metabolism	23	1.789057	0.002183	0.015492
Chagas disease (American trypanosomiasis)	71	1.564443	0.003033	0.021022
NOD-like receptor signaling pathway	42	1.590747	0.004124	0.027929
RNA polymerase	9	-2.49639	0.004695	0.03109
AGE-RAGE signaling pathway in diabetic complications	63	1.567282	0.005102	0.033052
Basal transcription factors	10	-2.00994	0.005348	0.0336
African trypanosomiasis	19	1.697458	0.005488	0.0336
Phototransduction	11	-1.79203	0.005525	0.0336
Mismatch repair	14	-2.40018	0.007042	0.041972
Pathogenic Escherichia coli infection	24	1.617733	0.007592	0.044083
Prion diseases	18	1.702542	0.007692	0.044083
Chemical carcinogenesis	21	1.636773	0.008929	0.048812
Epstein-Barr virus infection	102	1.433865	0.008991	0.048812
Ribosome biogenesis in eukaryotes	21	-2.48608	0.009009	0.048812
Homologous recombination	16	-2.13096	0.009259	0.048933
Glycine, serine and threonine metabolism	18	-1.8508	0.009524	0.048933
Nucleotide excision repair	18	-2.25299	0.009524	0.048933

Supplementary Table S2. Gene set enrichment analysis of IGF-1-associated DEGs in RNA seq

Enrichment gene set	Number of genes	NES	*p*. value	FDR
Cytokine-cytokine receptor interaction	138	2.279413	0.000999	0.007576
Chemokine signaling pathway	94	1.916094	0.001	0.007576
Osteoclast differentiation	76	2.101744	0.001004	0.007576
HTLV-I infection	94	1.608266	0.001005	0.007576
Cell adhesion molecules (CAMs)	67	2.078572	0.001007	0.007576
Tuberculosis	81	2.307253	0.001007	0.007576
Neuroactive ligand-receptor interaction	60	2.13638	0.001008	0.007576
Influenza A	63	1.895164	0.001008	0.007576
Phagosome	67	2.195085	0.001011	0.007576
Jak-STAT signaling pathway	58	1.754604	0.001013	0.007576
Herpes simplex infection	56	1.815455	0.001013	0.007576
Natural killer cell mediated cytotoxicity	62	1.667998	0.001014	0.007576
Leishmaniasis	48	2.156925	0.001014	0.007576
TNF signaling pathway	51	1.888268	0.001016	0.007576
Chagas disease (American trypanosomiasis)	52	2.02898	0.001017	0.007576
Toxoplasmosis	51	2.061357	0.001017	0.007576
Measles	52	1.90941	0.001017	0.007576
Rheumatoid arthritis	55	2.584394	0.001017	0.007576
T cell receptor signaling pathway	53	1.919773	0.001018	0.007576
Staphylococcus aureus infection	44	2.676677	0.001019	0.007576
NF-kappa B signaling pathway	53	1.967894	0.00102	0.007576
Toll-like receptor signaling pathway	49	2.096833	0.001024	0.007576
Hematopoietic cell lineage	49	2.492082	0.001025	0.007576
Pertussis	37	2.002893	0.001027	0.007576
Complement and coagulation cascades	39	1.956477	0.001029	0.007576
Platelet activation	39	1.77833	0.001029	0.007576
Intestinal immune network for IgA production	35	2.068319	0.001029	0.007576
Fc gamma R-mediated phagocytosis	42	1.626224	0.00103	0.007576
Systemic lupus erythematosus	37	2.447137	0.001031	0.007576
Inflammatory bowel disease (IBD)	41	2.211229	0.001035	0.007576
Salmonella infection	35	1.985182	0.001036	0.007576
Amoebiasis	39	1.974418	0.001037	0.007576
NOD-like receptor signaling pathway	33	1.985287	0.001038	0.007576
Malaria	34	1.927885	0.001045	0.007576
Allograft rejection	27	2.076045	0.001053	0.007576

Graft-versus-host disease	30	2.22679	0.001053	0.007576
Antigen processing and presentation	35	2.07405	0.001057	0.007576
Type I diabetes mellitus	27	2.240893	0.001062	0.007576
Autoimmune thyroid disease	26	2.120768	0.001062	0.007576
Primary immunodeficiency	23	2.114043	0.001072	0.007576
Asthma	21	2.206763	0.001076	0.007576
Legionellosis	27	2.026849	0.001082	0.007576
Prion diseases	13	2.006259	0.001151	0.007868
Viral myocarditis	30	1.94959	0.002101	0.013842
Inflammatory mediator regulation of TRP channels	33	1.668439	0.002119	0.013842
Leukocyte transendothelial migration	40	1.682727	0.003067	0.019605
PI3K-Akt signaling pathway	99	1.560282	0.004012	0.025097
Homologous recombination	7	-2.22232	0.004255	0.026059
African trypanosomiasis	22	1.726979	0.004343	0.026059
AGE-RAGE signaling pathway in diabetic complications	42	1.587015	0.005123	0.029715
Ribosome	7	-2.15131	0.005155	0.029715
B cell receptor signaling pathway	44	1.626247	0.006166	0.034864
DNA replication	12	-2.65596	0.006536	0.036176
Sulfur relay system	4	-1.74039	0.006645	0.036176
Arachidonic acid metabolism	14	1.72016	0.006826	0.036467
Transcriptional misregulation in cancer	66	1.515666	0.007049	0.036467
Calcium signaling pathway	45	1.569835	0.007128	0.036467
Fanconi anemia pathway	14	-3.13049	0.007194	0.036467
Terpenoid backbone biosynthesis	5	-1.93039	0.007491	0.037121
Aminoacyl-tRNA biosynthesis	13	-2.6785	0.007576	0.037121
Notch signaling pathway	16	-2.07943	0.008065	0.038513
Apoptosis	49	1.566251	0.008122	0.038513
RNA degradation	17	-2.06507	0.009709	0.045307
Epstein-Barr virus infection	61	1.492075	0.010132	0.046543
Cytosolic DNA-sensing pathway	19	1.650428	0.011038	0.048756
Rap1 signaling pathway	60	1.49359	0.011089	0.048756
Base excision repair	11	-1.96842	0.011111	0.048756

Supplementary Table S3. Differentially expressed miRNAs in TCGA database

miRNA	log2FC	*p*. value	FDR
hsa-miR-9	-0.89505488	9.91511E-09	7.56382E-07
hsa-miR-9*	-0.801860531	3.07865E-09	2.74E-07
hsa-miR-182	-0.520040014	0.00301651	0.019407427
hsa-miR-181d	-0.512222646	2.83765E-10	3.78826E-08
hsa-miR-130b	-0.458492634	2.92753E-07	1.737E-05
hsa-miR-204	-0.420631006	0.00983418	0.044884205
hsa-miR-149	-0.398294535	3.94079E-05	0.000861009
hsa-miR-181c	-0.376861655	2.64769E-06	0.000100991
hsa-miR-20a	-0.369237149	8.27248E-05	0.001472502
hsa-miR-17-5p	-0.350062953	1.48313E-05	0.000469051
hsa-miR-93	-0.344807768	1.22472E-06	5.45002E-05
hsa-miR-19b	-0.342828153	3.45563E-05	0.000802306
hsa-miR-183	-0.327498319	0.002036094	0.014306242
hsa-miR-106a	-0.32721886	0.000163754	0.002429024
hsa-miR-106b	-0.316848112	2.83898E-05	0.000689097
hsa-miR-19a	-0.315776869	0.000102707	0.001710394
hsa-miR-551b	-0.312841595	1.45087E-05	0.000469051
hsa-miR-25	-0.307063329	2.62907E-05	0.000668534
hsa-miR-33	-0.305311937	8.08515E-05	0.001472502
hsa-miR-301	-0.303310792	0.000873406	0.007737252
hsa-miR-15b	-0.28980843	0.000418426	0.004381168
hsa-let-7i	-0.252991091	0.00122461	0.009616786
hsa-miR-92	-0.251274319	0.000434037	0.004457222
hsa-miR-20b	-0.251075832	0.001858001	0.013228967
hsa-miR-125b	-0.238673545	0.001615363	0.01181649
hsa-miR-454-3p	-0.232472121	2.60653E-06	0.000100991
hsa-miR-181a	-0.222000188	0.003428138	0.021715185
hsa-miR-565	-0.220520743	0.004836833	0.028077236
hsa-miR-17-3p	-0.216336442	0.000963495	0.007915486
hsa-miR-95	-0.205201536	1.58386E-05	0.000469051
hsa-miR-181a*	-0.200559554	0.00037471	0.004148142
hsa-miR-130a	-0.184068668	0.004837277	0.028077236
hsa-miR-16	-0.182300565	0.010627856	0.0469031
hsa-miR-181b	-0.175851135	0.01032699	0.045955105
hsa-miR-30b	-0.175289214	0.006870202	0.034263948

hsa-miR-30d	-0.175188028	0.002766672	0.018467536
hsa-miR-32	-0.153638252	0.002521558	0.017262971
hsa-miR-550	-0.153462014	1.66879E-05	0.000469051
hsa-miR-450	-0.144794001	0.000212428	0.002941594
hsa-miR-542-3p	-0.128116856	0.006812065	0.034263948
hsa-miR-598	-0.115803631	0.000883843	0.007737252
hsa-miR-96	-0.10187845	0.004028603	0.024727287
hsa-miR-488	-0.084468135	0.001153848	0.009335675
hsa-miR-153	-0.076731623	0.000940341	0.007915486
hsa-miR-18b	-0.074718248	0.006982506	0.034263948
hsa-miR-182*	-0.062012709	0.001550489	0.01166974
hsa-miR-545	-0.032940408	0.007771547	0.037053627
hsa-miR-641	-0.030947909	7.18755E-05	0.001421537
hsa-miR-499	-0.027057869	8.167E-05	0.001472502
hsa-miR-579	-0.025427128	4.38262E-05	0.000900122
hsa-miR-302a*	-0.020533989	0.008298005	0.039213582
kshv-miR-K12-5	-0.020334362	0.000449893	0.00453288
ebv-miR-BART3-3p	-0.019297105	0.000523408	0.005081818
hsa-miR-597	-0.019007001	0.000225853	0.002941594
hsa-miR-580	-0.018941888	0.000625094	0.005856145
hsa-miR-573	-0.018415073	0.000321386	0.003730873
hsa-miR-559	-0.018382157	0.000372557	0.004148142
hsa-miR-556	-0.017960333	0.000956099	0.007915486
hcmv-miR-US25-2-3p	-0.01793193	0.003001006	0.019407427
hsa-miR-33b	-0.01786371	0.000200744	0.00289722
hsa-miR-633	-0.017682291	0.002963832	0.019407427
hcmv-miR-UL22A*	-0.01766213	0.000159874	0.002429024
hsa-miR-496	-0.016781127	0.000388403	0.004148142
hsa-miR-620	-0.01672721	0.004391412	0.026348471
hsa-miR-384	-0.016317267	0.001224114	0.009616786
hsa-miR-220	-0.016144928	0.007234846	0.034846307
hsa-miR-208	-0.015648167	0.003837745	0.023829717
hsa-miR-643	-0.015383828	0.000308152	0.003656738
hsa-miR-578	-0.015316231	0.005575525	0.030380921
hsa-miR-553	-0.015166803	0.005276751	0.02907767
kshv-miR-K12-9	-0.01496448	0.001551595	0.01166974
ebv-miR-BHRF1-3	-0.01492812	0.008693854	0.040369724

hsa-miR-515-5p	-0.014617979	0.006993952	0.034263948
hsa-miR-581	-0.014415158	0.005932817	0.031681241
hsa-miR-651	-0.013686581	0.003456537	0.021715185
hsa-miR-588	-0.012984329	0.006820564	0.034263948
hsa-miR-587	-0.012914183	0.005037457	0.028617045
ebv-miR-BART8-5p	-0.012662072	0.006779422	0.034263948
hsa-miR-802	-0.012340292	0.005153051	0.028965573
hsa-miR-570	-0.01181038	0.009967589	0.045107563
hsa-miR-380-5p	-0.011334969	0.007243333	0.034846307
ebv-miR-BART11-5p	-0.010769692	0.006787556	0.034263948

Supplementary Table S4. Differentially expressed miRNAs in IGF-1-treated U87-MG cells

microRNA	log2FC	*p*. value	FDR
hsa-miR-4286	-3.86340803	0.00119779	0.006247156
hsa-miR-21-5p	-3.776428643	0.000594439	0.004502231
hsa-miR-4324	-3.437377346	0.000307906	0.003782673
hsa-miR-125b-5p	-3.422284867	1.33495E-05	0.002682766
hsa-miR-1260a	-3.412556675	0.001571108	0.007518829
hsa-let-7f-5p	-3.366008733	0.00168036	0.007760399
hsa-miR-29a-3p	-3.304596296	6.35696E-05	0.003044482
hsa-miR-4284	-3.260275033	0.00055489	0.004486486
hsa-let-7a-5p	-3.232767769	0.000937372	0.005291837
hsa-let-7b-5p	-3.125524163	2.111E-05	0.002682766
hsa-miR-125a-5p	-3.123478916	0.000781895	0.005017908
hsa-let-7c	-3.032824745	0.000529879	0.004437741
hsa-miR-16-5p	-2.981460238	0.002514941	0.009735616
hsa-miR-100-5p	-2.973299209	0.000658154	0.004777104
hsa-let-7d-5p	-2.853225274	0.000181266	0.003432232
hsa-miR-363-5p	-2.738884166	0.016715958	0.031928946
hsa-miR-99a-5p	-2.72312379	0.001529296	0.007441435
hsa-miR-4443	-2.623173313	0.003651438	0.011867172
hsa-miR-221-3p	-2.595445936	0.00049295	0.004302863
hsa-miR-1260b	-2.586444374	0.001458975	0.007261525
hsa-miR-27a-3p	-2.481435069	0.002629342	0.009960573
hsa-let-7e-5p	-2.477183797	1.10477E-05	0.002682766
hsa-miR-23a-3p	-2.324290321	0.001012562	0.005581911
hsa-miR-222-3p	-2.305254565	0.002304791	0.009208592
hsa-miR-4454	-2.244617359	0.007240113	0.017713871
hsa-miR-3135b	-2.232321242	0.001044594	0.00568651
hsa-miR-23b-3p	-2.227350668	0.000176824	0.003422535
hsa-miR-29c-3p	-2.174050265	0.004426349	0.013155012
hsa-miR-29b-3p	-2.155169824	0.000267019	0.003666042
hsa-miR-26a-5p	-2.086770568	0.000262512	0.003666042
hsa-miR-9-5p	-2.076294635	8.09557E-05	0.003044482
hsa-miR-4448	-2.06797456	0.000196521	0.003461101
hsa-miR-98-5p	-2.054827895	2.2675E-05	0.002682766
hsa-miR-92b-3p	-2.017678006	0.000122348	0.003134271
hsa-miR-27b-3p	-1.960577536	0.000150992	0.003278479
hsa-miR-181a-5p	-1.935316814	0.002730164	0.010076154

hsa-miR-22-3p	-1.926914886	0.00351834	0.011520579
hsa-miR-5100	-1.926163956	0.001918716	0.008273277
hsa-let-7g-5p	-1.86730921	0.001566908	0.007518829
hsa-miR-92a-3p	-1.862715225	0.000566921	0.004486486
hsa-miR-10a-5p	-1.794456723	0.000832806	0.005181244
hsa-miR-19b-3p	-1.782090645	0.003221341	0.010709113
hsa-miR-15a-5p	-1.766947629	1.28634E-05	0.002682766
hsa-miR-4632-5p	-1.706269151	0.001421664	0.007147281
hsa-miR-3676-5p	-1.687153286	0.002194069	0.00888853
hsa-miR-17-5p	-1.680264168	5.6506E-05	0.003044482
hsa-miR-34a-5p	-1.650560493	0.001083722	0.005862867
hsa-miR-20a-5p	-1.644807137	0.000188268	0.003461101
hsa-miR-99b-5p	-1.641297333	0.001739499	0.007932481
hsa-miR-138-5p	-1.621375455	4.3139E-05	0.003044482
hsa-miR-4701-5p	-1.553542613	0.000319387	0.003782673
hsa-miR-20b-5p	-1.544410202	0.002829985	0.010102117
hsa-miR-195-5p	-1.521233384	0.000310382	0.003782673
hsa-miR-24-3p	-1.515682691	0.00532294	0.014718353
hsa-miR-541-3p	-1.497100677	0.003058193	0.010410386
hsa-miR-4521	-1.485060678	0.01421783	0.029138188
hsa-miR-19a-3p	-1.440796452	0.001766095	0.008011818
hsa-miR-26b-5p	-1.399364803	7.49382E-05	0.003044482
hsa-miR-106a-5p	-1.390104092	0.000563079	0.004486486
hsa-miR-4695-5p	-1.383013147	0.000716373	0.004935933
hsa-miR-15b-5p	-1.382991225	0.000273585	0.003666042
hsa-miR-3607-5p	-1.352913327	3.97826E-05	0.003044482
hsa-miR-30d-5p	-1.335354945	1.78517E-05	0.002682766
hsa-miR-3676-3p	-1.31081031	0.000925161	0.005291837
hsa-miR-4633-5p	-1.306500155	0.008395189	0.019816286
hsa-miR-193b-3p	-1.294100567	0.005295496	0.014718353
hsa-miR-30a-5p	-1.287130103	0.001610843	0.007584025
hsa-miR-3651	-1.271590325	0.019060474	0.035322708
hsa-miR-766-3p	-1.240387232	0.001111117	0.005929484
hsa-miR-181b-5p	-1.228963832	0.000633946	0.004679385
hsa-miR-34b-5p	-1.224791916	0.005053764	0.014239154
hsa-miR-130a-3p	-1.222573236	3.75034E-05	0.003044482
hsa-miR-197-3p	-1.209233768	0.002156279	0.008829628
hsa-let-7i-5p	-1.190042164	0.00158933	0.007523403

hsa-miR-30e-5p	-1.181722778	0.006101931	0.015817804
hsa-miR-145-5p	-1.177426322	0.000694282	0.004916417
hsa-miR-4776-5p	-1.170651667	0.000255127	0.003666042
hsa-miR-1294	-1.154041341	0.006162205	0.015926648
hsa-miR-4649-5p	-1.110780785	0.000152854	0.003278479
hsa-miR-30b-5p	-1.110637914	0.005465431	0.014876219
hsa-miR-3665	-1.080360439	0.002883697	0.010159519
hsa-miR-4723-5p	-1.077372548	0.014815888	0.029688384
hsa-miR-4763-5p	-1.076093049	0.002906113	0.01016556
hsa-miR-668	-1.074891955	0.017841505	0.033563608
hsa-miR-4323	-1.065304906	0.001939305	0.008302711
hsa-miR-10b-5p	-1.050837786	0.000255807	0.003666042
hsa-miR-106b-5p	-1.050182677	9.92011E-05	0.003044482
hsa-miR-6511b-5p	-1.04229023	0.000348389	0.003782673
hsa-miR-933	-1.030042271	0.001692854	0.007760399
hsa-miR-181c-5p	-1.02915592	9.99939E-05	0.003044482
hsa-miR-3607-3p	-1.023779257	0.00180969	0.00812495
hsa-miR-129-1-3p	-1.00853249	0.00272957	0.010076154
hsa-miR-423-3p	-0.999612575	0.000742373	0.004935933
hsa-miR-634	-0.981519145	0.000455007	0.004181275
hsa-miR-1234-5p	-0.973339061	0.007024008	0.017364068
hsa-miR-4664-5p	-0.970607605	0.001845455	0.008136079
hsa-miR-129-2-3p	-0.96533947	0.002802625	0.010102117
hsa-miR-1248	-0.962132704	0.000586471	0.004486486
hsa-miR-4313	-0.945802359	0.015983994	0.031145546
hsa-miR-191-5p	-0.943374766	0.000115348	0.003044482
hsa-miR-4695-3p	-0.940192413	0.008739506	0.020244972
hsa-miR-151a-5p, hsa-miR-151b	-0.933713802	7.00968E-05	0.003044482
hsa-miR-30c-5p	-0.931248238	0.00694074	0.017223317
hsa-miR-21-3p	-0.927805045	0.000309794	0.003782673
hsa-miR-1229-3p	-0.920418573	0.010480369	0.023194434
hsa-miR-455-3p	-0.919666023	0.000356444	0.003782673
hsa-miR-93-5p	-0.915111792	0.000329055	0.003782673
hsa-miR-107	-0.913596023	0.000937067	0.005291837
hsa-miR-449b-3p	-0.908959091	0.010701698	0.02353833
hsa-miR-28-5p	-0.904848914	0.000192747	0.003461101
hsa-miR-25-3p	-0.89108089	0.00036433	0.003782673
hsa-miR-3622a-3p	-0.862554327	0.000148741	0.003278479

hsa-miR-3620-3p	-0.844918513	0.00456786	0.013351028
hsa-miR-3176	-0.822848895	0.003775917	0.012135881
hsa-miR-1976	-0.822697181	0.000145405	0.003278479
hsa-miR-1538	-0.818393194	4.08586E-05	0.003044482
hsa-miR-4655-5p	-0.795534442	0.000250059	0.003666042
hsa-miR-103a-3p	-0.774756177	0.003715618	0.011986307
hsa-miR-718	-0.769012381	0.019727158	0.036249692
hsa-miR-4534	-0.764419313	0.006531566	0.01639317
hsa-miR-6511a-5p, hsa-miR-6511b-5p	-0.757325951	0.004352418	0.012982728
hsa-miR-9-3p	-0.752434052	8.58655E-05	0.003044482
hsa-miR-1234-3p	-0.743050216	0.000755242	0.004951375
hsa-miR-4780	-0.711759781	0.001189597	0.006241803
hsa-miR-181d	-0.690381932	0.000898423	0.005287342
hsa-miR-1193	-0.685310254	0.007275196	0.017749317
hsa-miR-4687-5p	-0.684400183	0.030037741	0.049739302
hsa-miR-6511a-3p	-0.674797011	0.004988558	0.014199458
hsa-miR-3621	-0.670900494	0.000198685	0.003461101
hsa-miR-130b-3p	-0.665803971	0.000108169	0.003044482
hsa-miR-6131	-0.661022776	0.000722941	0.004935933
hsa-miR-4258	-0.653112689	0.008226694	0.019577734
hsa-miR-5739	-0.651765914	0.00643948	0.016210368
hsa-miR-4728-3p	-0.651722036	0.00268536	0.009995506
hsa-miR-374a-5p	-0.649438381	0.021036835	0.038014695
hsa-miR-361-5p	-0.641717555	0.00032352	0.003782673
hsa-miR-5010-5p	-0.637630204	0.002681718	0.009995506
hsa-miR-143-3p	-0.633588298	0.003819925	0.012187381
hsa-miR-4253	-0.632515152	0.000224486	0.003620873
hsa-miR-4713-5p	-0.629579768	0.004713903	0.013640562
hsa-miR-1290	-0.623682249	0.004554033	0.013351028
hsa-miR-4758-3p	-0.619041329	0.004509433	0.013290062
hsa-miR-3180-5p	-0.617191311	0.002002636	0.008427568
hsa-miR-4723-3p	-0.611246243	0.004252651	0.012816812
hsa-miR-4446-3p	-0.593379927	0.008340213	0.019740014
hsa-miR-424-5p	-0.590286123	0.003990323	0.012547188
hsa-miR-125b-1-3p	-0.588281227	0.016645633	0.031864498
hsa-miR-320d	-0.55929349	0.00180614	0.00812495
hsa-miR-3184-3p	-0.557391432	0.005515287	0.014965155
hsa-miR-6072	-0.557389738	0.003587862	0.011704225

hsa-miR-1238-3p	-0.555987226	0.000247445	0.003666042
hsa-miR-1292-3p	-0.554908761	0.000674578	0.004826312
hsa-miR-3182	-0.549418348	0.009965084	0.022295263
hsa-miR-6717-5p	-0.542129695	0.000215362	0.003615686
hsa-miR-4722-3p	-0.535923066	0.011851756	0.025363341
hsa-miR-4455	-0.532698485	0.021301591	0.038413427
hsa-miR-4290	-0.513171182	0.00130108	0.006666124
hsa-miR-6509-3p	-0.510409745	0.000502405	0.004332622
hsa-miR-22-5p	-0.502375895	0.011913453	0.025432885
hsa-miR-4712-3p	-0.498820364	0.000407546	0.004033778
hsa-miR-574-3p	-0.498290747	0.000726974	0.004935933
hsa-miR-3196	-0.497596786	0.00031416	0.003782673
hsa-miR-1228-3p	-0.496275031	0.004440383	0.013155012
hsa-miR-1224-3p	-0.491207551	0.001999564	0.008427568
hsa-miR-708-5p	-0.487975565	0.000646589	0.004732598
hsa-miR-4750-3p	-0.486881063	0.002548677	0.009779286
hsa-miR-18a-5p	-0.475485356	0.007502064	0.018145201
hsa-miR-3145-5p	-0.468845049	0.001684294	0.007760399
hsa-miR-3149	-0.45889939	0.020217869	0.036763598
hsa-miR-210	-0.457755238	0.00026337	0.003666042
hsa-miR-4499	-0.457191003	0.015707211	0.030882576
hsa-miR-3972	-0.45489879	0.000840685	0.005193167
hsa-miR-466	-0.448831614	0.000558884	0.004486486
hsa-miR-1233-3p	-0.4369562	0.002827816	0.010102117
hsa-miR-378a-3p	-0.43364479	0.000620368	0.004618297
hsa-miR-664a-3p	-0.43042866	0.02904339	0.048452726
hsa-miR-3940-3p	-0.425767816	0.000884713	0.005253452
hsa-miR-4769-3p	-0.420174651	0.011143967	0.024326805
hsa-miR-422a	-0.411697467	0.02689241	0.045659433
hsa-miR-484	-0.399465674	0.003921822	0.012421479
hsa-miR-1181	-0.39216564	0.004757449	0.013720988
hsa-miR-4301	-0.391026742	0.025180886	0.043344965
hsa-miR-2116-3p	-0.390983859	0.00887405	0.020447877
hsa-miR-378g	-0.389762788	0.000580157	0.004486486
hsa-miR-3622b-5p	-0.387109378	0.022099032	0.039202152
hsa-miR-486-5p	-0.383129963	0.000456052	0.004181275
hsa-miR-186-5p	-0.382360931	0.00279372	0.010102117
hsa-miR-4725-5p	-0.380189406	0.005791083	0.015616203

hsa-miR-374b-5p	-0.374718178	0.000738983	0.004935933
hsa-miR-151a-3p	-0.374718178	0.000738983	0.004935933
hsa-miR-3173-5p	-0.370731155	0.000479555	0.004262166
hsa-miR-335-5p	-0.369550669	0.000545326	0.004486486
hsa-miR-4646-3p	-0.357950598	0.004235161	0.012808422
hsa-miR-3174	-0.357941898	0.000564857	0.004486486
hsa-miR-532-3p	-0.357928492	0.016225484	0.031270783
hsa-miR-365a-3p, hsa-miR-365b-3p	-0.356710442	0.003976656	0.012547188
hsa-miR-6722-3p	-0.35403017	0.002794441	0.010102117
hsa-miR-885-3p	-0.352658815	0.005972197	0.015636783
hsa-miR-3177-3p	-0.351964688	0.002159255	0.008829628
hsa-miR-6124	-0.350543679	0.001907736	0.008266858
hsa-miR-4436b-5p	-0.341380847	0.019522084	0.035971118
hsa-miR-4429	-0.33821777	0.012543126	0.026646494
hsa-miR-378i	-0.331780048	0.004895105	0.013979135
hsa-miR-4787-3p	-0.331482506	0.001031467	0.005650363
hsa-miR-4456	-0.329640672	0.00654974	0.01639317
hsa-miR-146b-5p	-0.325158119	0.003368177	0.011112433
hsa-miR-320e	-0.323957349	0.027826867	0.046880467
hsa-miR-29b-1-5p	-0.323267737	0.002792004	0.010102117
hsa-miR-1470	-0.322663127	0.01474776	0.029688384
hsa-miR-140-3p	-0.319891532	0.001103411	0.005929484
hsa-miR-18b-5p	-0.314995307	0.001944607	0.008302711
hsa-miR-320a	-0.314157806	0.000878231	0.005253452
hsa-miR-6125	-0.310746289	0.001900939	0.008266858
hsa-miR-4717-5p	-0.302209895	0.004868512	0.013948928
hsa-miR-5193	-0.300626883	0.002105413	0.008750386
hsa-miR-6511b-3p	-0.297716559	0.022178812	0.03926371
hsa-miR-625-5p	-0.297217468	0.007330019	0.017783973
hsa-miR-339-5p	-0.291632856	0.002854139	0.010146755
hsa-miR-142-3p	-0.287058421	0.001185492	0.006241803
hsa-miR-664b-3p	-0.284720114	0.002681304	0.009995506
hsa-miR-378c	-0.28163533	0.004043	0.012621695
hsa-miR-125b-2-3p	-0.279853041	0.014816836	0.029688384
hsa-miR-221-5p	-0.273183512	0.002543223	0.009779286
hsa-miR-491-3p	-0.26781382	0.006213297	0.015979272
hsa-miR-6716-3p	-0.261223008	0.005432673	0.014876219
hsa-miR-101-3p	-0.260618452	0.011742411	0.025191231

hsa-miR-887	-0.257388825	0.020435013	0.037081033
hsa-miR-1225-3p	-0.254073737	0.005957564	0.015636783
hsa-miR-23c	-0.252474854	0.026299415	0.044827379
hsa-miR-4685-3p	-0.244029769	0.013380352	0.027946464
hsa-miR-6508-5p	-0.24348429	0.021471728	0.038498649
hsa-miR-378f	-0.221954673	0.008485657	0.019868299
hsa-miR-378e	-0.219060556	0.003054888	0.010410386
hsa-miR-1236-3p	-0.213997019	0.021828909	0.03888135
hsa-miR-6514-3p	-0.213990801	0.005067896	0.014239154
hsa-miR-877-3p	-0.207381942	0.016192364	0.031270783
hsa-let-7a-3p	-0.20166135	0.006349106	0.01607579
hsa-miR-7-5p	-0.187983287	0.005448599	0.014876219
hsa-miR-132-3p	-0.185301761	0.005369633	0.014753786
hsa-miR-4745-5p	-0.182819703	0.005858856	0.015636783
hsa-miR-1914-5p	-0.175848869	0.006435813	0.016210368
hsa-miR-378d	-0.16411938	0.005570158	0.015067105
hsa-miR-345-5p	-0.161368051	0.007182186	0.017621645
hsa-miR-3146	-0.158814942	0.014812483	0.029688384
hsa-miR-302c-5p	-0.15266972	0.013541072	0.028014901
hsa-miR-5089-3p	-0.139658877	0.016263679	0.031270783
hsa-miR-185-5p	-0.134905408	0.01616727	0.031270783
hsa-miR-129-5p	-0.134408379	0.015344666	0.030375463
hsa-miR-148b-3p	-0.132206828	0.024503095	0.042514335
hsa-miR-1227-3p	-0.126303139	0.016261188	0.031270783
hsa-miR-503-5p	-0.111563714	0.023518202	0.041216003
hsa-miR-4641	-0.111034673	0.020683933	0.037454689
hsa-miR-3166	-0.096756258	0.030191723	0.049899413

Supplementary Table S5. The correlation analysis between miRNAs and genes of cytokine and cytokine receptor interaction

Gene	Correlation	*p* value
**miR-9-5p-trageted genes**		
IL1R2	-0.36738	< E-16
TGFBR2	-0.33934	1.78E-15
CCR1	-0.33128	9.33E-15
CXCL9	-0.1928	9.72E-06
GHR	-0.0964	0.028096
**miR-9-3p-trageted genes**		
IL10RA	-0.35876	<E-16
TGFBR2	-0.32266	4.86E-14
CSF2RB	-0.27791	1.17E-10
GHR	-0.08798	0.045135
**miR-130b-trageted genes**		
TGFBR2	-0.49218	3.55E-15

Supplementary Table S6. Statistical calculations for high IGF-1/low miRNA status

Covariate	Hazard Ratio	95% Conf-U	95% Conf-L	stdErr	*p*. value
**High IGF-1/Low miR-9-5p status**					
High IGF-1/ Low miR-9-5p	1.224	1.672	0.896	0.159	0.203
G-CIMP	2.786	5.372	1.445	0.335	0.002
Age > 60	1.362	1.867	0.993	0.155	0.054
Karnofsky score ≥ 80	2.457	3.469	1.740	0.176	<0.001
Gender (male)	1.054	1.437	0.773	0.158	0.748
**High IGF-1/Low miR-9-3p status**					
High IGF-1/ Low miR-9-3p	1.456	2.028	1.045	0.169	0.026
G-CIMP	2.695	5.777	1.257	0.389	0.011
Age > 60	1.274	1.733	0.937	0.157	0.124
Karnofsky score ≥ 80	2.012	2.886	1.403	0.184	<0.001
Gender (male)	1.023	1.428	0.733	0.170	0.978
**High IGF-1/Low miR-130b status**					
High IGF-1/ Low miR-130b	1.24	1.707	0.901	0.163	0.187
G-CIMP	4.38	8.82	2.175	0.357	<0.001
Age > 60	1.941	2.745	1.372	0.177	<0.001
Karnofsky score ≥ 80	1.756	2.514	1.226	0.183	0.002
Gender (male)	0.908	1.253	0.658	0.164	0.556

Supplementary Table S7. Survival analysis of miR-181d- or miR-9-3p-targeted genes

Gene	Target miRNA	Hazard Ratio	SD of log intensities	*p* value
IL10RA	miR-181d, miR-9-3p	1.111	1.129	0.0123275
CSF2RB	miR-181d, miR-9-3p	1.144	0.895	0.0142532
IL7R	miR-181d	1.078	0.948	0.0860837
TGFBR2	miR-181d, miR-9-3p	1.086	0.68	0.239493
IL1R1	miR-181d	1.07	0.626	0.382742
CXCL9	miR-181d	1.029	1.398	0.426054
IL2RB	miR-181d	1.066	0.57	0.438288
GHR	miR-181d, miR-9-3p	0.971	1.038	0.549928
CXCL12	miR-181d	0.976	1.032	0.631965

SD of log intensities : standard deviation of the log 2 of the gene expression level

Supplementary Table S8. Primer list

Primer name	Sequence
**For miR-181d gene cloning**	
miR-181d-clon-F	ACAGAATTCAACATTCAACCTGTCGGTGAG
miR-181d-clon-R	ACAGGATCCCCAACCCTCTAATATTTAGTC
**For 3’UTR cloning**	
CCR1-3UTR-F	TATCTCGAGGACCATAGGAGGCCAACCCA
CCR1-3UTR-R	ATCTCTAGAATTCCAAGAGCCCAAATCCT
IL1B -3UTR-F	TATCTCGAGTGTGCTGAATGTGGACTCAATCCCTAGGGCTG
IL1B -3UTR-R	ATCTCTAGACTTCAGTGAAGTTTATTTCAGAACCA
**For 3’UTR Mutagenesis**	
CCR1-3UMUT-F	ACTTGGGATAGAGAGGGCCGACAATGGTGGCCTGGGGCTT
CCR1-3UMUT-R	AAGCCCCAGGCCACCATTGTCGGCCCTCTCTATCCCAAGT
IL1B -3UMUT-F	TATCTCGAGTGTGCTGCCGACGGACTCAATCCCTAGGGCTG
**For real-time PCR**	
IL1B -sybr-F	ATGATGGCTTATTACAGTGGCAA
IL1B -sybr-R	GTCGGAGATTCGTAGCTGGA
CCR1-sybr-F	GACTATGACACGACCACAGAGT
CCR1-sybr-R	CCAACCAGGCCAATGACAAATA
GAPDH-sybr-F	GTG AAG GTC GGA GTC AAC
GAPDH-sybr-R	GTT GAG GTC AAT GAA GGG

Supplementary Figure S1

A

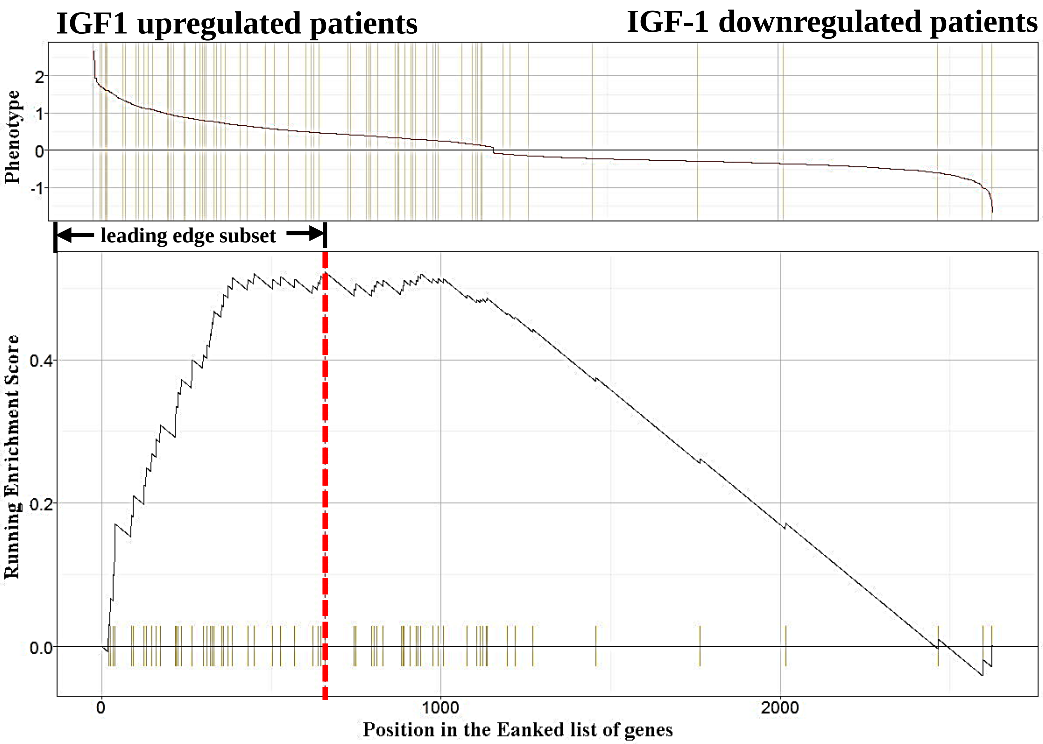

B

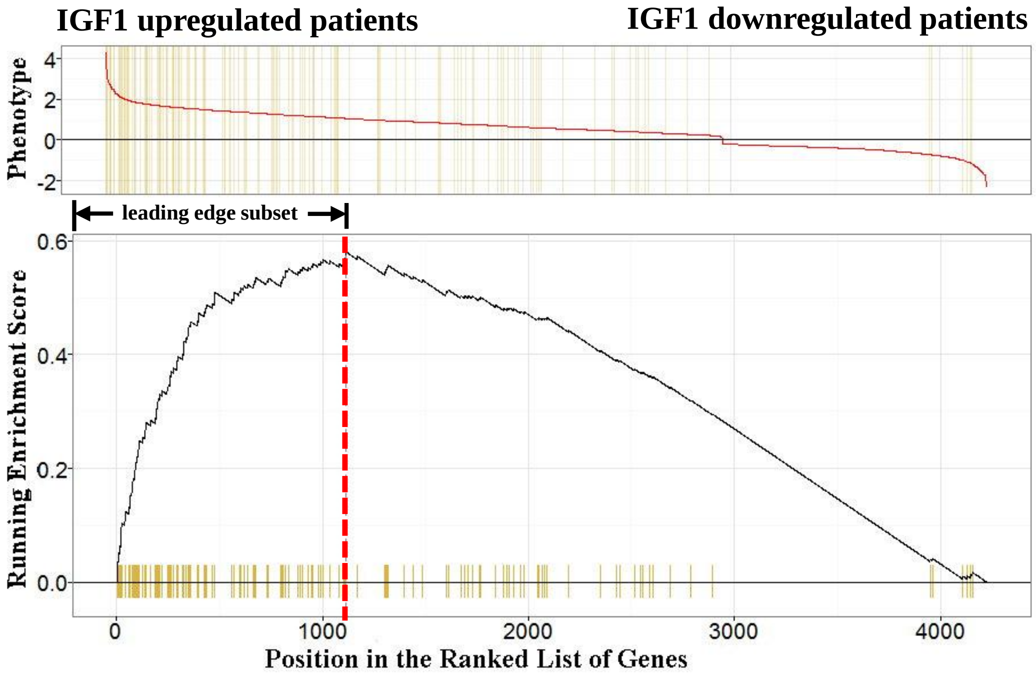

Supplementary Figure S2

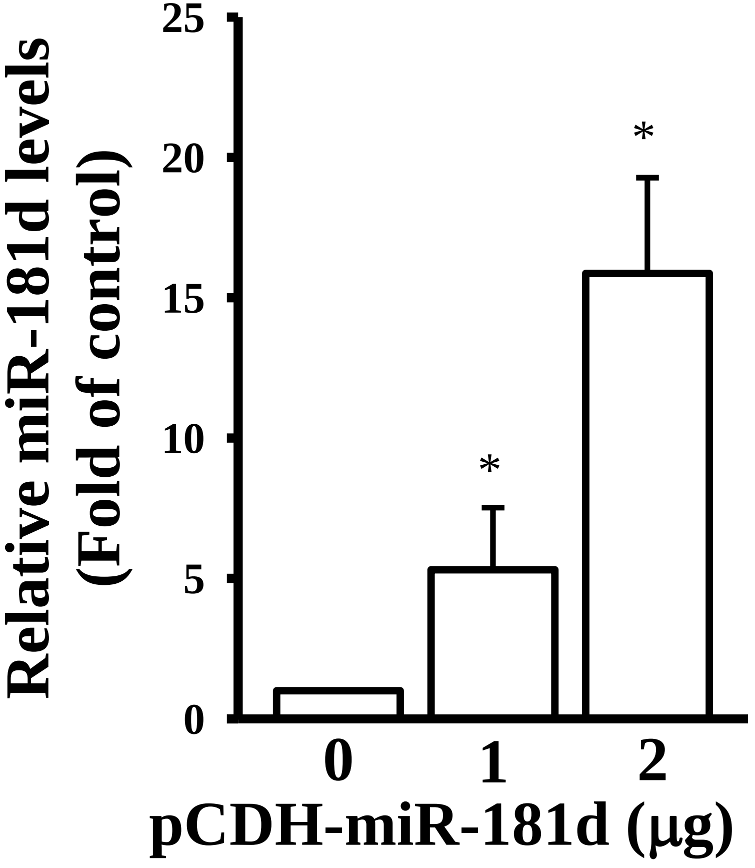

Supplementary Figure S3

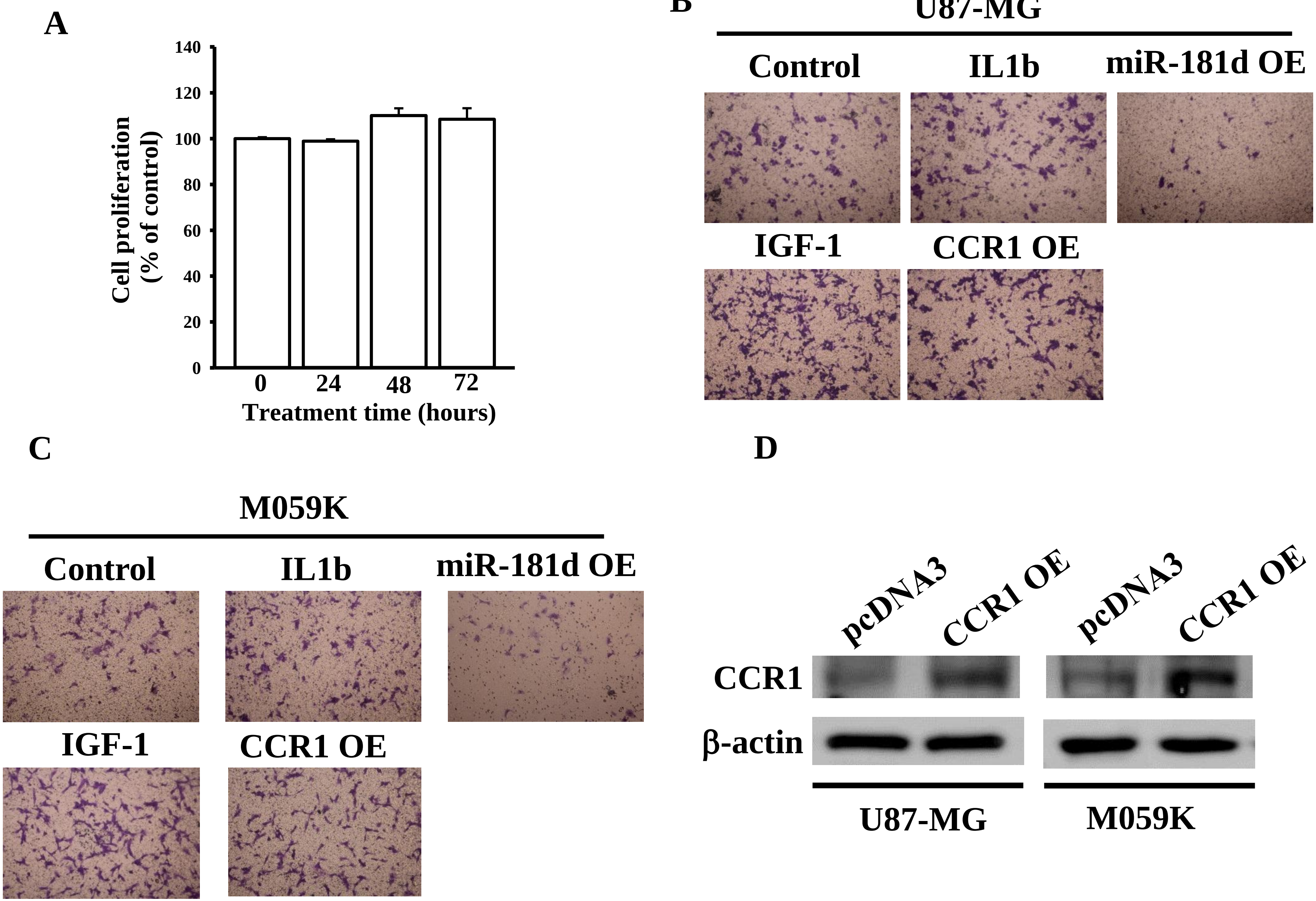

Supplement: Supplementary file 2 — Supplementary figure and Table [file 41598_2017_826_MOESM2_ESM.pdf]
